# Students' Interactional Cultural Capital and Academic Performance in Test‐ and Teacher‐Based Assessments

**DOI:** 10.1111/1468-4446.13199

**Published:** 2025-03-07

**Authors:** Sara Geven, Dieuwke Zwier

**Affiliations:** ^1^ Department of Sociology University of Amsterdam Amsterdam the Netherlands; ^2^ Department of Political and Social Sciences (SPS) European University Institute (EUI) Florence Italy

**Keywords:** cultural capital, educational inequality, school performance, teacher bias, track recommendations

## Abstract

Qualitative work highlights the significance of students' interactional cultural capital in educational settings—that is, cultural resources that help to navigate/interact with educational institutions and gatekeepers. We make a first attempt to measure expressions of students' interactional cultural capital quantitatively, and examine their relationship with academic performance. Using data on over 1200 Dutch students in their final year of primary school, we find positive associations between several expressions of students' interactional cultural capital (knowledge about the educational system; perceived cultural match between home and school) and academic performance. These positive relationships are equally strong for teacher‐ and test‐based assessments of performance, suggesting that these forms of cultural capital help students in their learning rather than providing educational benefits via teacher biases. We find little support for positive relations between students' help‐seeking strategies and academic performance. Different help‐seeking behaviors do not form a unified cultural “strategy” and are not stratified by socio‐economic status (SES) as anticipated. For educational knowledge, we find some support for the cultural mobility hypothesis: SES‐based performance gaps, particularly in teacher assessments, are smaller among students with greater knowledge of the educational system.

## 1 Introduction

The concept of cultural capital has become indispensable in research on social inequality in industrialized societies ever since Bourdieu's (1986) introduction of it (e.g., Calarco 2018; Friedman and Laurison 2020). Following Bourdieu's original work, many scholars focus on cultural capital in educational settings, particularly pointing at differences in the cultural resources that parents and students of different socio‐economic status (SES) bring to the classroom (Davies and Rizk 2018; Jæger 2022; Lareau 2015).

Despite its omnipresence in explanations for educational inequalities, cultural capital remains an ambiguous concept without a clearly agreed‐upon definition. This seems to be partly due to the fact that the concept is employed by a wide range of scholars working from different research traditions that tend to “talk past one another” (Davies and Rizk 2018). Quantitative studies mostly try to capture cultural capital by focusing on preferences for, and participation in, high culture, such as reading habits, musical preferences, and museum or theater visits (Davies and Rizk 2018; Lareau and Weininger 2003; Van der Waal et al. 2024). While they tend to find (small) positive relationships between cultural capital and educational outcomes, critics allude that educational gatekeepers primarily reward other forms and expressions of culture. This critique is grounded in qualitative work highlighting the importance of daily, micro‐interactional, and oftentimes more subtle manifestations of culture for educational outcomes (Davies and Rizk 2018; Gaddis and Murphy 2022; Lareau 2015; Lareau and Weininger 2003; Streib 2011). In this literature, cultural capital is defined as the strategies, knowledge, and skills that enable the successful interaction with, and navigation of, educational gatekeepers and institutions. It includes for example parents' strategies to intervene in school matters (Lareau 2015) or children's help‐seeking behavior in class (Calarco 2011).

Despite important findings in qualitative work, attempts to re‐shift or broaden the conception and operationalization of cultural capital in quantitative research are limited. Some studies widen their survey‐based measures of cultural capital by incorporating aspects of parenting styles (e.g., Davies and Rizk 2018). Other research has introduced new methods for tapping into “non‐declarative” cultural capital through implicit associations with various types of cultural consumption (Van der Waal et al. 2024). Yet, children's *own* manifestations of cultural knowledge and strategies in school are hardly considered (cf., Calarco 2011; Harvey 2022; Streib 2011). While qualitative studies may be better suited to capture subtle interactional forms of cultural capital (Davies and Rizk 2018; Gaddis and Murphy 2022), they may be less equipped to measure (differences in) *amounts* of cultural capital and shed light on the *extent* to which cultural capital relates to educational outcomes (Gaddis and Murphy 2022). Hence, we know relatively little about which expressions of students' interactional cultural capital are relevant in the educational sphere.

This paper contributes to existing work by making a first attempt to quantitatively study how children's own manifestations of interactional cultural capital relate to educational performance. We examine both teacher‐ and test‐based assessments of performance. In doing so, we hope to shed light on the potential mechanism linking (interactional) cultural capital to educational outcomes. The literature has proposed two mechanisms: teacher biases and academic skills. The first implies that cultural capital is misconceived as academic aptness or allows students to meet the non‐neutral academic expectations set by teachers. According to the second mechanism, cultural capital relates to educational benefits, because it captures skills that boost student learning, not only in the eyes of the teacher (Breinholt and Jæger 2020). Previous quantitative studies mainly support this latter mechanism (e.g., Breinholt and Jæger 2020; Dumais 2006; Roscigno and Ainsworth‐Darnell 1999; for a review see Jæger 2022). However, these studies have not yet examined interactional cultural capital, for which the “teacher bias” mechanism may be particularly salient. Moreover, some prior studies only test whether cultural capital relates to either teacher‐ *or* test‐assessed performance (Jæger 2022), and not its relationship to both. Using Seemingly Unrelated Regressions (SURs), we analyze these two performance measures *jointly*, and formally test how indicators of students' interactional cultural capital relate to teacher‐assessed performance *in comparison to* test‐assessed performance.

Our study relies on new linked survey‐register data from students in their final year of primary school (age 11–12) in the Netherlands, a setting where cultural capital is arguably of particularly relevant. Specifically, students are about to be allocated to separate educational programs based on their academic performance (i.e., between‐school ability tracking). Primary school teachers play a crucial role in this allocation: they form binding track recommendations for each student, which are highly consequential for students' educational careers (Geven 2024). In this context, students may be particularly incentivized to activate cultural resources to meet teachers' expectations and/or enhance their test performance. Simultaneously, this context may enhance teachers' reliance on cultural resources in their assessments (Jæger 2022). Teachers likely face considerable insecurity in their allocation task, as they have to formulate long‐term educational expectations for young students. This insecurity may cause them to rely on traits that (are believed to) proxy for academic potential (Geven et al. 2021), including students' cultural resources.

The Dutch context is also ideal for comparing teacher‐ and test‐assessed performance: Dutch primary school teachers provide a track recommendation just before students take a final “track recommendation” test. Hence, teachers' overall assessment of a student can be well compared to a similar test‐based assessment. Moreover, instead of using performance assessments collected solely for research purposes, we rely on assessments that have high stakes for both teachers and students.

## 2 Theory

### 2.1 Interactional Cultural Capital and Educational Outcomes

Work on cultural capital has its roots in Bourdieu's cultural reproduction theory. According to this, cultural resources constitute a non‐material form of capital that—similarly to economic and social resources—can be converted into other types of benefits (Bourdieu 1986; Bourdieu and Passeron 1990). Cultural capital generally pertains to the acquaintance with the cultural codes dominant in a particular setting, also described as “knowing the rules of the game” (Lareau 2015; Lareau and Weininger 2003; Van der Waal et al. 2024). Cultural capital is typically acquired through family socialization, and students from different social backgrounds vary in their cultural capital. More specifically, the socio‐economic conditions that students are raised in, shape their cognitively ingrained dispositions, perceptions, and habits (i.e., habitus) (Bourdieu and Passeron 1990). The habitus can be understood as the cultural capital that can be mobilized in particular settings or situations (i.e., fields): the more the habitus aligns with the expectations or requirements for success that are prevalent in a given field, the more cultural capital a person has (Edgerton and Roberts 2014, 207). Schools are believed to be middle‐class institutions whose cultural codes are implicitly ingrained with the culture that children from advantaged SES backgrounds are typically socialized with (Calarco 2018; Lareau 2015). Hence, students from advantaged SES backgrounds would possess more cultural capital conducive of school success.

Studies on cultural capital and educational outcomes focus on different forms, or manifestations of, cultural capital. Inspired by DiMaggio (1982), quantitative research has primarily examined parents' or students' appreciation or consumption of “high‐status” culture, such as preferences for classical music and literature, reading activities, or theater and museum visits (Davies and Rizk 2018). Recent research aiming to quantitatively capture “non‐declarative” cultural capital continues to focus on cultural consumption in its operationalization (Van der Waal et al. 2024). Following Lareau (2000), qualitative work highlights forms of cultural capital that allow for the effective interaction with, and navigation of, educational institutions and gatekeepers (Calarco 2018; Harvey 2022; Lareau 2015). We refer to these cultural strategies, knowledge, and skills/mannerisms as interactional cultural capital.

Originally, work on interactional cultural capital has focused on *parents*, including their ability to negotiate with educational gatekeepers or the strategies they use to intervene in school matters; which require cultural knowledge of how educational institutions work in the first place (Lareau 2015). More recent qualitative work suggests that children's *own* interactional cultural resources may also impact school success. For example, students vary in their help‐seeking requests from teachers, and those who are more assertive seem to be rewarded in school (Calarco 2018). Similarly, children seem to differ, from a young age, in their linguistic styles (Streib 2011) and acquaintance with the bodily mannerisms expected in school, such as greetings or maintaining eye contact with teachers (Harvey 2022). Such differences could translate into educational inequalities, not only because failing to comply with school standards may be (implicitly) sanctioned by teachers, but also because students who feel more at ease in school may be better able to focus on school tasks (Stephens et al. 2012).

**FIGURE 1:**
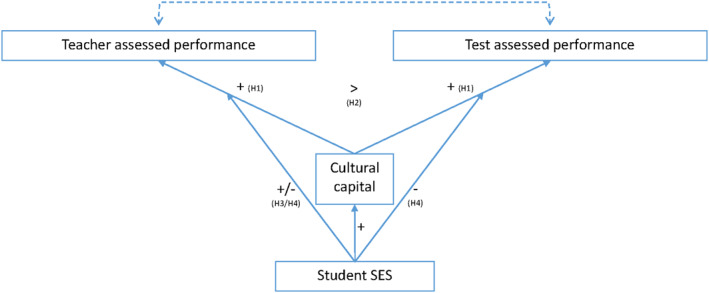
Theoretical model.

Building on this recent qualitative work, we quantitatively study students' interactional cultural capital. We consider three different manifestations of it: (1) students' perceived cultural alignment between the mannerisms prevalent at home and in school (“cultural match”), (2) the strategies they employ to seek help from their teacher (Calarco 2011, 2018), and (3) students' knowledge of the educational system (Lareau 2015). We hypothesize (see Figure 1):

H 1 *Students with higher levels of interactional cultural capital (i.e., who experience more alignment between school and home mannerisms, are more knowledgeable about the educational system, and are more assertive in seeking help from teachers) perform better in school.*

### 2.2 Mechanisms That Link Cultural Capital to Educational Outcomes

Recent quantitative work proposes two mechanisms via which children can convert their cultural capital into educational benefits (Breinholt and Jæger 2020; Jæger 2022). The first, known as the “teacher bias” mechanism, has its roots in Bourdieu's cultural reproduction theory (Breinholt and Jæger 2020). According to this, certain cultural resources—for example, accents, mannerisms, skills and/or preferences—are rewarded by educational gatekeepers, as they are misperceived as academic aptness or brilliance (Breinholt and Jæger 2020; DiMaggio 1982; Jæger 2022). Students with more cultural capital have an easier time to live up to the expectations of educational institutions, even if they are not more academically talented. The second mechanism operates *through* students' academic competencies. Cultural capital would capture skills like creativity, analytical thinking, and linguistic proficiency that improve learning (Breinholt and Jæger 2020; Jæger 2022). For example, engagement in arts and music may enhance creativity, and thereby also (traditional) academic competencies, such as cognitive reasoning.

Children's interactional cultural capital can translate into educational benefits via both channels. First, students may receive educational rewards via teacher biases, independent of their actual (improved) understanding of the material. For example, assertive help‐seekers may make fewer mistakes and work faster because they received (more hints for) the correct answer. While their flawless assessments may cause teachers to perceive them as more competent, they may not perform better in situations where they cannot ask for help or are not assessed by their teachers. Moreover, teachers may arbitrarily value assertive help‐seeking as an important academic skill (i.e. valuing pro‐active learners; Calarco 2011) and reward this, for example, by placing students in gifted programs or higher ability tracks. Other forms of interactional cultural capital, such as the way students speak or their knowledge of the educational system, may also translate into educational benefits in this way: while not necessarily related to academic competencies, they may be misperceived as such.

Second, children's interactional cultural capital may allow students to learn more (effectively). For example, students who experience a “cultural match” between school and home may have higher levels of academic self‐efficacy conducive to learning (Jæger 2022). In contrast, students who experience a cultural mismatch have to worry about “appropriate” ways of acting which may distract from school tasks (Stephens et al. 2012). Similarly, students' knowledge of educational institutions may enhance their learning by helping students understand how to study for tests or identifying which tests or selection moments to prioritize (Forster and van de Werfhorst 2020). Also, the strategies that students employ in teacher interactions can enhance learning opportunities (Jæger 2022). For example, students with more assertive help‐seeking strategies may receive more explanation from their teacher, and therefore develop a better understanding of the material.

So far, quantitative studies have found most support for the second mechanism (i.e., cultural capital operating via skills rather than teacher bias; Jæger 2022; Breinholt and Jæger 2020). However, past studies have focused on aspects of cultural capital that involve children's cultural participation or preferences. Expressions of children's interactional cultural capital have not yet been considered, and it is possible that teacher biases *do* play an important role in their conversion. The educational advantages associated with such interactional forms are by nature more dependent on the perception, recognition, and actions of educational gatekeepers. This especially applies to manifestations that directly involve educational gatekeepers. For example, the extent to which children's help‐seeking strategies or cultural mannerisms translate into educational rewards is contingent on the extent to which teachers (implicitly) recognize, act upon, and reward them. Although they may indirectly also enhance test‐based performance (e.g., through receiving more explanation or attention from the teacher), direct effects on test results are expected to be smaller, as standardized tests are in theory blind to these strategies and mannerisms. We hypothesize (see Figure 1):

H 2 *Students' forms of cultural capital that involve interactions with educational gatekeepers (i.e., experiencing more alignment between home and school mannerisms and being more assertive in seeking help from teachers) are more positively related to teacher‐than to test‐assessed student performance.*

### 2.3 Cultural Mobility or Cultural Reproduction

There is an ongoing debate about the role of cultural capital in educational inequalities. While traditionally, cultural capital was believed to (re)produce SES inequality in education (i.e., cultural reproduction hypothesis), later work proposed that students from disadvantaged background may employ cultural capital for upward social mobility (i.e., cultural mobility hypothesis; DiMaggio 1982; Jæger 2022).

According to the first perspective, cultural capital is socially‐stratified: students from advantaged SES backgrounds generally possess more cultural resources valued in educational institutions (Bourdieu 1986; Bourdieu and Passeron 1990). Students primarily acquire cultural capital through parental socialization in early childhood, making it relatively stable over the life course. Cultural capital returns are also larger for students from advantaged SES backgrounds. Since children from disadvantaged backgrounds lack the (early) family socialization of cultural capital, they will never be able to completely embody upper middle‐class culture and reap its benefits. In line with this, research shows how students from disadvantaged class backgrounds have difficulties to become fully part of upper middle class cultures, even after having gone through the university system themselves (Friedman and Laurison 2020). If cultural resources are recognized or valued less when students from disadvantaged backgrounds possess them, this should be especially apparent in performance assessments involving educational gatekeepers, and less so in standardized (multiple‐choice) tests. Hence, we hypothesize the following with respect to *teacher‐*assessed performance (see Figure 1):

H 3 *The positive relationship between students' interactional cultural capital and teacher‐assessed performance is stronger for students from advantaged SES backgrounds.*

In contrast to the cultural reproduction hypothesis, DiMaggio (1982) introduced the cultural mobility hypothesis, arguing that status groups have become more fluid in modern societies. While cultural capital still signals familiarity with high status culture, it has become less associated with a specific SES or class background. Students from advantaged backgrounds are socialized with more cultural capital relevant in educational settings in their childhood, yet cultural capital is not a fixed trait and can be acquired outside of the family (e.g., in school) throughout the life course. More importantly, students from disadvantaged backgrounds may employ high status cultural resources for upward social mobility, implying that returns to cultural capital may be higher for them (DiMaggio 1982). First, cultural capital could compensate for the typically lower levels of other educational resources available at home. For example, lower SES parents may lack (cultural) knowledge and skills needed to effectively interact with educational gatekeepers or help children prepare for important tests (Van Rooijen et al. 2019). Students who possess more interactional cultural capital may be able to successfully interact with educational gatekeepers and institutions *themselves* (e.g., negotiate higher track recommendations, know how to (adequately) study for high stakes tests). Conversely, students from advantaged SES backgrounds are less in need of these forms of cultural capital, as they can rely on their parents. Second, students from disadvantaged backgrounds with more interactional cultural capital may suffer less from (implicit) SES‐based discrimination. Teachers may be less likely to perceive them as coming from a disadvantaged background, and this could reduce the activation of negative SES‐based academic stereotypes (Klapproth, Kärchner, and Glock 2018).

Based on the cultural mobility hypothesis, we thus expect greater educational returns from cultural capital for students from disadvantaged SES backgrounds. While one of the underlying arguments primarily applies to teacher‐assessed performance (i.e., reduction of SES‐based discrimination), the idea that students' cultural capital may compensate for a lack of educational resources at home also applies to test‐based assessments. We hypothesize:

H 4 *The positive relationships between students' interactional cultural capital and their performance in teacher‐ and test‐based assessments are stronger for students from disadvantaged SES backgrounds.*

## 3 Dutch Educational Context

The Dutch educational system is typically classified as a between‐school tracking context. After primary school (grade 6; age 11–12), students are separated into entirely different educational tracks on the basis of their academic performance (Geven 2024). There are six tracks: a track for students with special educational needs (pro), three pre‐vocational tracks (vmbo‐b, vmbo‐k, vmbo‐(g)t), an intermediary track (havo), and an academic track providing direct entry to university (vwo). Student allocation to these tracks is determined by a binding recommendation, following from both teacher‐ and test‐based performance assessments. Primary school teachers provide a track recommendation for all grade‐6 students. While teachers are expected to consider longitudinal performance data from the school's administrative system (LVS, *Leerling Volg Systeem* in Dutch), they have substantial discretion in how to weigh (other) factors ‐ including student traits beyond (prior) performance ‐ in their recommendation. After teachers have formulated an initial track recommendation, students take a final standardized test that also provides a track recommendation, independent of the teacher. Schools can choose between five different expert‐approved standardized multiple‐choice tests. All tests provide raw scores on different subjects, an overarching raw score, and a track recommendation. In case the test's recommendation exceeds that of the teacher, teachers are required to reconsider their initial recommendation. Teachers can—but are not required to—make *upward* adjustments.^1^ Despite efforts to monitor and standardize track recommendations, track recommendations remain to be lower for equally performing students from disadvantaged SES backgrounds (Geven 2024).

## 4 Data

We use data from the PRIMS project (an acronym for transition from Primary to Secondary school), specifically designed to study students' transition from primary to secondary school in the Netherlands (Zwier et al. 2023). We use data from the first wave of the second cohort (February‐March 2021), including information on students' cultural capital in the final year of primary school (grade 6). The online surveys were administered before students participated in the final standardized test. Most students had already received their teacher's initial tracking recommendation.

Data were collected following a two‐stage sampling procedure. First, primary schools were sampled from all Dutch regular primary schools. Larger schools and schools with a higher share of students from disadvantaged SES backgrounds were oversampled. The 79 participating schools are representative of regular Dutch primary schools with respect to region, urbanization level, SES composition, denomination and the track recommendation students received. In the second stage, all grade‐6 students at these schools were invited to participate, and 64% obtained active consent from their parents or caregivers to do so. Students from non‐European minority backgrounds and those without tertiary‐educated parents or from lower income households are slightly underrepresented in the data (reference blinded).

In total, 1411 grade‐6 students completed (part of) the survey, of which 1378 could be linked to the Netherlands Cohort Study on Education (in Dutch: *Nationaal Cohortonderzoek Onderwijs* [NCO]; see Haelermans et al. 2020). NCO includes administrative data on teachers' initial and final track recommendations, students' final test scores, and parental SES and migration background. After removal of cases with missing data, the sample includes 1248 students in 107 classes in 79 schools (8 students had a missing on one of the dependent variables, 122 additional students had (a) missing on (an) independent variable(s)).

For a subsample of students, we have information on prior (grade‐5) test performance in reading and mathematics. In 2020, the Netherlands Initiative for Education Research approached all Dutch school boards to make data from their administration systems (LVS) available for research purposes. About 25% of the Dutch student population are included in these data. From our sample, 362 students could be matched to these data.

## 5 Measures

### 5.1 Dependent Variables

We measure teacher‐assessed performance by teachers' track recommendation prior to students' participation in the final test. This information is obtained from the Dutch registers. Teachers could recommend students to six tracks, or a combination of two adjacent tracks: pro, pro/vmbo‐b, vmbo‐b, vmbo‐b/k, vmbo‐k, vmbo‐k/(g)t, vmbo‐(g)t, vmbo‐(g)t/havo, havo, havo/vwo, vwo. Since the tracks are hierarchically ordered, this 11‐point scale can be treated as a continuous measure (see Timmermans, Boer, and Werf 2016). We standardize the scale to compare teacher‐to test‐assessed performance.

We obtain the score on the final standardized test in primary school from the Dutch registers to measure test‐assessed performance. Schools could opt between five tests that all provide subject‐specific scores, an overall score, and a track recommendation. While teacher track recommendations are on an 11‐point scale, test‐based recommendations are on a six‐point scale, covering all adjacent‐track combinations (pro/vmbo‐b, vmbo‐b/k, vmbo‐k/(g)t, vmbo‐(g)t/havo, havo/vwo) and the academic track (vwo). Despite this different scale, teachers can use the overall test score to discern which of the two adjacent tracks best matches a student's performance. Since the overall score includes more detailed performance information, we use this measure—transformed to standard‐normal scores^2^—rather than the test‐based track recommendation.

### 5.2 Independent Variables

The data include three expressions of interactional cultural capital. Students' experienced cultural alignment between home and school is measured with three items that capture the extent to which students feel that: (1) at home, they talk completely differently to one another than the teachers talk to them at school, (2) teachers use completely different words than parent(s)/caregivers, and (3) they have to be completely different at school than at home (*Difference home‐school)*. These items are adjusted from items in the National Educational Panel Study (NEPS), starting cohort 2. We calculate an average score, ranging from fully disagree (0) to fully agree (4), as items load on one factor and form a reliable scale (*α* = 0.74 in the full dataset; reference blinded).

Students' *knowledge about the educational system* is measured with five questions about potential pathways in the educational system, such as whether it is possible to enter university directly after obtaining an intermediary track secondary school degree (also see Forster and van de Werfhorst 2020). We calculate the total number of correct answers (i.e., 0–5 range). In case students indicated they did not know an answer, this is treated as incorrect.

*Students' help‐seeking strategies* form a third type of interactional cultural capital. Based on Calarco's (2011, 2018) work, four items were developed to tap into different help‐seeking behaviors. Students were asked if they would ask their teacher for help (e.g., by raising their hand) in case: (1) they do not understand a question on a test (*test help‐seeking*), the teacher explains something to the class and they do not understand it (*public help‐seeking*), (3) they want to know whether they did well on an exercise (*affirmative help‐seeking*), (4) they want help but the teacher is busy doing something else (*assertive help‐seeking*). As these items do not form a reliable scale (*α* = 0.56), we consider each item separately. Answers range from fully disagree (0) to fully agree (4).

Parental socio‐economic status (*SES*) is measured by a dummy indicating whether at least one of the parents has obtained a Bachelor degree (ISCED 5–8). We obtain this from the Dutch registers where people's attainment of university and university of applied sciences certificates have been registered since, respectively, 1983 and 1986. The attainment of other tertiary certificates is not registered for the full population, yet we assume that parents born in the Netherlands after 1966 did not complete a Bachelor degree (see Zwier and Geven 2023).

We perform supplementary analyses using two alternative SES measures. First, we measure parental education with three categories: parents without a Bachelor degree (ISCED 0–4), parent(s) with a Bachelor degree (ISCED 5–6), and parent(s) with a Master's degree or higher (ISCED 7–8) (Supporting Information S1: Table A7). Second, we perform analyses using a natural logarithm of the disposable equivalized household income of the parent with whom the child is living (Supporting Information S1: Table A8). This measure is corrected for household size and composition with the equivalence scale of Statistics Netherlands.

### 5.3 Control Variables

For both outcomes (i.e., teacher‐ and test‐assessed performance), we control for a student's age, self‐identified gender, and a dummy indicating whether a student has a minority background (i.e., at least one of the parents is born outside of either Europe (excluding Turkey), the United States, Oceania, Indonesia, or Japan).^3^ Moreover, for test‐assessed performance, we include a categorical variable indicating which standardized test was used. Although we use standard‐normal scores, the selection of tests by schools is non‐random (Expertgroep toetsing PO 2018).

For a subsample (*N* = 362), we control for prior performance in standardized reading and mathematics tests. We use mid‐term scores from 2019/2020, which were administered 1 year before our survey data were collected. We standardize test scores using all available scores from students in the same grade.

Supporting Information S1: Table A1 provides descriptive statistics for all variables for the full and the subsample (for which prior performance data are available). For most variables, the subsample's proportion/mean does not statistically significantly differ from the full sample, except for assertive help seeking and the type of final standardized test administered. Supporting Information S1: Table A2 shows correlations between all variables included in the analyses.

## 6 Analytical Strategy

We start our analyses by examining the extent to which students of different SES backgrounds differ on the interactional cultural capital measures. Subsequently, we use Seemingly Unrelated Regressions (SURs) within the Systemfit package in R to jointly predict teacher‐ and test‐assessed student performance (Henningsen and Hamann 2007).^4^

Prior studies have usually regressed cultural capital indicators on teacher‐ or test‐assessed performance while controlling for, respectively, test‐ or teacher‐assessments (e.g., Breinholt and Jæger 2020; Dumais 2006; Roscigno and Ainsworth‐Darnell 1999; see Jæger 2022). Some studies focus on one assessment type, and do not explicitly test whether cultural capital relates to *both* (e.g., Dumais 2006). Other studies predict the different types in *separate* models (e.g. Breinholt and Jæger 2020), thereby assuming that the error terms of the outcomes are uncorrelated. Since it is likely that these outcomes share common unobserved predictors (e.g., IQ), we use SURs. This approach allows for statistical tests to compare estimates across outcomes, while accounting for the correlation in the models' error terms (Zellner 1962).

Our SUR approach also responds to a recently raised issue concerning studies on teacher bias (Van Huizen, Jacobs, and Oosterveen 2024). Teacher bias is often operationalized as the difference in teacher assessment for students with different background traits (e.g., SES, cultural capital) after controlling for students' test scores as a measure of “true” scholastic ability. This approach implicitly assumes that test scores perfectly reflect ability, and fails to account for measurement error (e.g., students may have a bad day). Consequently, the true impact of students' ability on teacher assessment is underestimated, inflating the estimates of student traits that are positively correlated with ability (e.g., SES, cultural capital). In our SUR approach, we operationalize teacher bias (hypothesis 2) as the extent to which cultural capital indicators (or SES) are more positively related to teacher‐assessed performance than to test‐assessed performance. Since test‐assessed performance is treated as a dependent variable, it is not assumed to be free of measurement error. While we focus on SURs, we compare SUR findings to findings based on the analytical approach of prior quantitative studies on cultural capital and school performance (i.e., multi‐level models predicting teacher‐assessed performance while accounting for test‐assessed performance and vice versa (Supporting Information S1: Tables A9,A10).

We first estimate a model in which we only include SES and the control variables, and no cultural capital measures. To test relationships between students' interactional cultural capital and performance (hypothesis 1), we estimate separate models for each of our three cultural capital aspects (i.e., *Difference home‐school*, *knowledge about the educational system*, and *help‐seeking behaviors*).^5^ We conduct joint χ^2^ tests to test the hypothesis that the relationships to teacher‐ *and* test‐based performance are different from zero. To examine whether our cultural capital measures are more strongly related to teacher‐than test‐assessed performance (hypothesis 2), we test the equivalence of estimates across both outcomes with a χ^2^ test. Finally, we assess whether relationships between cultural capital and performance vary by SES (hypotheses 3 and 4) by including interaction terms. We calculate clustered standard errors, robust to the clustering of students in classes (Arai 2015).^6^

We estimate all SUR models on the full sample (*N* = 1248) and the subsample for whom we have information on prior performance (*N* = 362). For the subsample, we predict models with and without prior performance measures. We include prior performance to partly account for students' academic ability, which may confound the relations between cultural capital and academic performance. While we measure academic ability with error (van Huizen, Jacobs, and Oosterveen 2024), controlling for prior performance may actually lead to an underestimation of the cultural capital estimates in these relations: estimates will discard the potential positive impact that cultural capital had on prior performance.

## 7 Results

### 7.1 Descriptive Results

**FIGURE 2:**
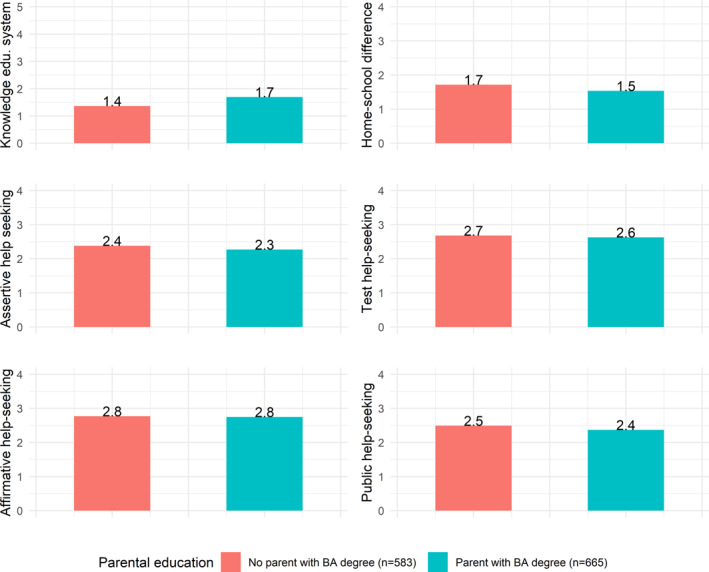
Descriptive statistics, by parental education.

Figure 2 shows the average scores on the interactional cultural capital indicators by parental education. We find statistically significant differences in students' perceived home‐school difference and educational knowledge by parental education (home‐school difference: *t* (1217.5) = 3.32; *p* < 0.01; educational knowledge (*t* (1237.9) = −4.039; *p* < 0.01), with students with (a) parent(s) with a BA‐degree scoring higher on these indicators. However, contrary to expectations based on cultural reproduction theory (Bourdieu 1986; Bourdieu and Passeron 1990), differences are small. Students whose parents do not hold a BA degree have on average 0.3 less correct answers on the six educational knowledge questions, and score 0.2 points lower on the five‐point home‐school difference scale (i.e., both 0.21 of a standard deviation).

We also find SES gaps in students' assertive and public help‐seeking (assertive: *t* (1221.3) = 1.98, *p* = 0.05; public: *t* (1213.3) = 2.03, *p* = 0.04). However, in contrast to Calarco's (2011, 2018) findings, students with (a) parent(s) with a BA‐degree score slightly *lower* on these behaviors than students without parents with a BA‐degree (i.e., around 0.1 of a standard deviation). There are no SES differences in the other two help‐seeking behaviors.

### 7.2 Regression Results

**FIGURE 3:**
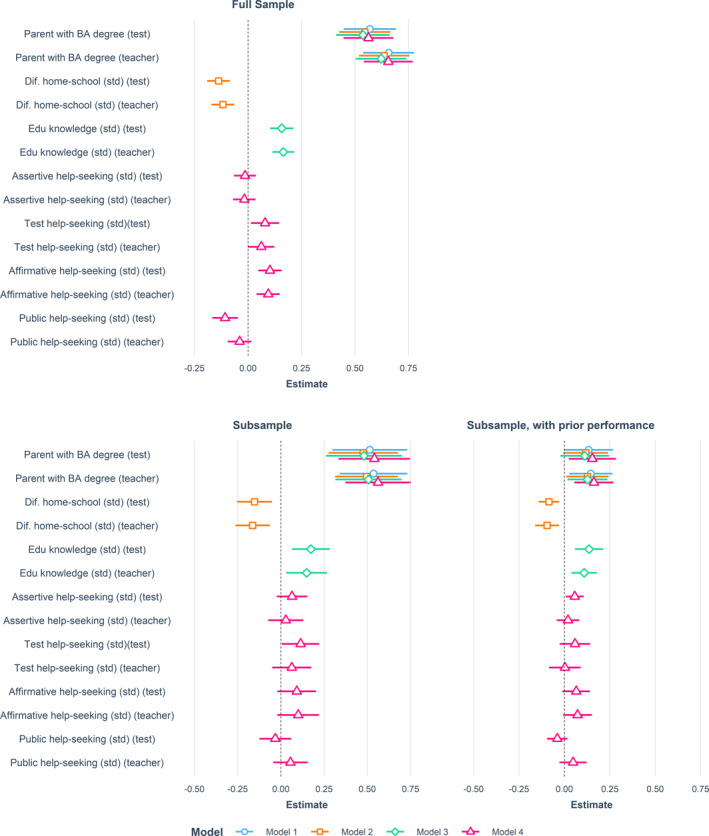
SUR estimates of teacher and test‐based assessments of student performance, without and with accounting for students' prior performance in grade 5. Estimates are based on models including all control variables. The upper panel shows the estimates of models without controlling for students' performance in grade 5, using the full sample (*n* = 1248, for all model estimates, see Supporting Information S1: Table A1). The bottom panel shows estimates of models using the subsample, without (left) and with accounting for students' performance in grade 5 (right) (*n* = 362 for all models, see Supporting Information S1: Table A2 and A3).

Figure 3 summarizes the SUR findings. The left panels show the estimates of models that do not account for prior performance for the full‐ (Supporting Information S1: Table A3, upper panel) and subsample (Supporting Information S1: Table A4, bottom panel). The right panel shows estimates for the subsample that control for prior performance (Supporting Information S1: Table A5). We present results for the subsample without prior performance to verify that differences in the results stem from the inclusion of prior performance, rather than potential differences in sample size or selectivity. When discussing the findings of models without prior performance, we focus on those using the full sample.

Model 1 only includes parental education and the control variables, and no cultural capital indicators. Parental education is positively related to test‐ and teacher‐based performance (joint test: *χ* ^2^ (2) = 117.91, *p* < 0.01 without prior performance; *χ* ^2^ (2) = 6.91, *p* = 0.03 with prior performance). In the full sample, the relationship is slightly stronger for teacher‐than test‐assessed performance (difference of 0.087; *χ* ^2^ (1) = 5.09, *p* = 0.02). In line with prior studies on SES biases in teacher assessments (Batruch et al. 2023), this indicates that teacher‐assessed performance is more stratified by SES than standardized‐test assessments. This difference does not reach significance in the subsample (without prior performance: *χ* ^2^ (1) = 0.12, *p* = 0.73; with prior performance: *χ* ^2^ (1) = 0.03, *p* = 0.86).

In models 2–4 we test relations between our cultural capital indicators and student performance (hypothesis 1). We find support for our hypothesis when considering students' perceived home‐school differences (model 2) and educational knowledge (model 3). In the full sample, a one‐standard‐deviation increase in perceived home‐school difference is related to, respectively, a 0.14 and 0.12 standard deviation lower test‐ and teacher‐assessed performance. These estimates are 0.09 and 0.10 in models accounting for prior performance. A one‐standard‐deviation increase in educational knowledge is related to an 0.16 (full sample) and 0.14 (subsample, with prior performance) higher test‐assessed performance score, and an 0.17 (full sample) and 0.11 (subsample, with prior performance) higher teacher‐assessed performance. The found relationships exist over and above the association between parental education and performance. Interestingly, parental education estimates remain highly stable across the models, suggesting that SES‐based performance gaps are not explained by SES differences in perceived home‐school differences or educational knowledge.

There is little support for hypothesis 1 when considering help‐seeking. While there are statistically significant relationships between help‐seeking and performance (joint test: *χ* ^2^ (8) = 44.13, *p* < 0.01 without prior performance; joint test: *χ* ^2^ (8) = 27.06, *p* < 0.01 with prior performance), findings are less equivocal and robust. When prior performance is not accounted for, there is a negative relationship between public help‐seeking and test‐based performance, contradicting hypothesis 1. Possibly, lower performing students ask more questions in class. Affirmative and test help‐seeking are, as expected, positively related to test‐ and teacher‐based performance (standardized estimate of, respectively, ±0.10 and ± 0.07 for both outcomes). However, when accounting for prior performance, none of the help‐seeking behaviors are positively related to the performance outcomes, except for assertive help‐seeking.

One possible explanation is that educational rewards associated with help‐seeking are conditional on *how* students make help requests (Harvey 2022). Only students who do so in the “appropriate,” “expected” manner may reap benefits. Similarly, students may reap more benefits from their educational knowledge if they know better *how* to interact with educational gatekeepers. We perform additional analyses to assess whether help‐seeking behaviors or educational knowledge relate more positively to educational performance for students experiencing more alignment between home and school. We find no support for this (Supporting Information S1: Table A6).

In contrast to hypothesis 2, we find little support that cultural capital relates more positively to teacher‐than test‐assessed performance. Only public help‐seeking is more positively related to teacher‐than test‐assessed performance (full sample: *χ* ^2^ (1) = 9.50, *p* = 0.002; subsample with prior performance: *χ* ^2^ (1) = 5.82, *p* = 0.02). However, its relation with teacher‐assessed performance is non‐significant. It is possible that teachers give lower assessments to low test performers, yet (slightly) higher assessments to students who ask questions in class. If low performers ask more questions, these effects cancel out.

**FIGURE 4:**
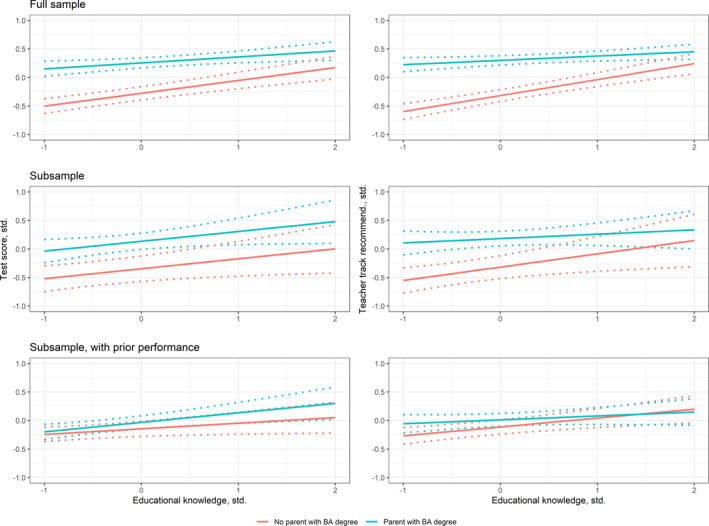
Interaction between educational knowledge and SES on test‐based (left) and teacher‐based (right) assessment of performance, without and with accounting for prior performance. Top‐panel predictions are based on a model without controlling for prior student performance, using the full sample (*n* = 665 Parent with BA‐degree, *N* = 583 No parent with BA degree, see model 6, Supporting Information S1: Table A1). Middle‐panel predictions are based on a model without accounting for prior student performance, using the subsample (*n* = 160 parent with a BA‐degree, *n* = 202 no parent with BA‐degree, see model 6, Supporting Information S1: Table A2). Bottom‐panel predictions are based on a model accounting for prior student performance, using the subsample (*n* = 160 parent with a BA‐degree, *n* = 202 no parent with BA‐degree, see model 5, Supporting Information S1: Table A3).

In a final set of models, we test SES heterogeneity in the relationships between cultural capital and performance. In contrast to hypothesis 3 and 4, there are no statistically significant interactions between parental education and either students' perceived home‐school difference or help‐seeking (models 5 and 7 in Supporting Information S1: Tables A3–A5). In line with hypothesis 4 (i.e., cultural mobility), we find a significant *negative* interaction between parental education and students' educational knowledge in the full sample (Supporting Information S1: Table A3, model 6; joint test *χ* ^2^ (2) = 16.01, *p* < 0.01). This implies that SES‐based performance gaps are smaller among students with more knowledge of the educational system. The upper two panels of Figure 4 show this interaction for test‐ and teacher‐assessed performance. While high SES students outperform low SES students at lower levels of educational knowledge, this advantage disappears at higher levels of educational knowledge. The reduction in the SES gap is particularly pronounced for teacher‐assessed performance, where the interaction is also significantly stronger (*χ* ^2^ (1) = 5.18, *p* = 0.02). This implies that the SES bias in teacher assessments is weaker among students with higher levels of educational knowledge.

In the subsample, we find no significant interaction between parental education and educational knowledge (joint *χ* ^2^ test: *p* = 0.186 without prior performance; *p* = 0.207 with prior performance). For teacher‐assessed performance, the pattern of the interaction remains negative, resulting in a smaller SES gap among students with higher levels of educational knowledge (Figure 4, right middle and bottom panel). While findings suggest that the relation between educational knowledge and (teacher‐assessed) performance is conditional on measures of parental education (see also Supporting Information S1: Table A7), we do not find that this relation is conditional on household income (Supporting Information S1: Table A8).

### 7.3 Comparison to Alternative Analytical Approach

We compare our SUR findings to results from a more commonly used analytical approach in prior studies: multilevel models predicting teacher‐assessed performance while accounting for test‐assessed performance (Supporting Information S1: Table A9) and vice versa (Supporting Information S1: Table A10). Consistent with our SUR findings, we find positive relationships between interactional cultural capital and student performance (hypothesis 1). Given students' test performance, teacher‐assessed performance is higher for students who have more knowledge of the educational system, engage in more public help‐seeking, and experience less home‐school differences, although the latter just not reaches statistical significance (*p* = 0.09). Conditioning on teacher‐assessed performance, experienced home‐school differences are negatively related to the test‐assessed performance of students whose parents do not hold a Bachelor degree (Supporting Information S1: Table A10, model 5).

The multilevel models mostly support relationships between cultural capital indicators and teacher‐assessed performance (conditioning on test‐assessed performance), suggesting that interactional cultural capital primarily operates via teacher biases rather than enhancing students' test performance beyond teacher assessments (hypothesis 2). Our SUR findings did not provide support for this. This discrepancy may stem from the overestimation of teacher bias in the multilevel models, which do not account for potential measurement error in test performance (van Huizen, Jacobs, and Oosterveen 2024). Similarly, the multilevel models show an SES “bias” in teacher assessments of 0.17 (i.e., SES estimate in table A9, model 1), nearly double the size of what we find in the SURs (i.e., 0.09; difference between SES estimates for teacher‐ and test‐assessed performance in Supporting Information S1: Table A3, model 1).

Similarly to the SURs, the multilevel models show a negative interaction between students' educational knowledge and parental SES for teacher‐assessed performance (hypothesis 4). This suggests that the SES bias in teacher assessments is smaller for students with greater educational knowledge.

## 8 Conclusions

Qualitative work suggests that children differ in the extent to which they possess interactional cultural capital relevant to educational settings. Children from advantaged SES backgrounds seem to hold cultural strategies, knowledge, and skills that ease their interactions with, and/or navigation of, educational gatekeepers and institutions. For example, they are more acquainted with the expected mannerisms in school, and have effective strategies at their disposal to receive help from teachers (also see Calarco 2011; Harvey 2022). While scholars argue that students' interactional cultural capital may contribute to (SES inequality in) performance, so far no quantitative studies have assessed (1) the extent to which students from different SES backgrounds vary in manifestations of their interactional cultural capital, and (2) how strongly these manifestations relate to academic performance in teacher‐ and test‐based assessments.

This paper made a first attempt to examine this by focusing on three manifestations of students' own interactional cultural capital: (1) knowledge about the educational system, (2) perceived differences between school and home mannerisms (i.e., “cultural match”), and (3) strategies used to seek help from teachers. We found that students from more advantaged SES backgrounds (1) were generally slightly more knowledgeable about the educational system and (2) experienced fewer differences between home and school mannerisms. Moreover, both cultural capital indicators were positively related to teacher‐ and test‐assessed school performance.

Building on work by Calarco (2011, 2018) in the United States, we anticipated that students from different SES backgrounds would also employ different help‐seeking “strategies,” consisting of a set of interrelated behaviors. Students from advantage backgrounds would make more requests for different types of help, which would benefit them academically. Interestingly, in our study, different help‐seeking behaviors did not form a reliable scale, and did not vary by SES as anticipated. Moreover, we found little support for positive relationships between separate help‐seeking behaviors and student performance. These findings may imply that, in the Netherlands, help‐seeking behaviors do not comprise a unified cultural *strategy* that is stratified by SES. Potentially, this is also *because* assertive help‐seeking is less rewarded in this context. Another possibility is that it is difficult to capture the subtleties of students' help‐seeking behavior with quantitative (self‐report) measures. Relatedly, SES differences may be more apparent in *how* students ask for help, rather than how often they report to do so (Harvey 2022). For example, students from disadvantaged backgrounds may perform assertive help requests in a less “successful” way. In supplementary analyses, we tested whether help‐seeking behaviors were more positively related to performance for students experiencing a greater “cultural match,” yet found no support for this. Since this is the first study to capture students' help‐seeking behaviors quantitatively, and relate these to school performance, we advise caution in drawing definitive conclusions and encourage future studies to incorporate similar measures, also in other contexts.

We found little support for the hypothesis that students' interactional cultural capital is more positively related to teacher‐than test‐assessed performance, with an exception for educational knowledge for students' without college‐educated parents. This lack of support may imply that interactional cultural capital does not merely enhance performance by being misrecognized as academic brilliance by teachers, but also boosts learning or skills that enhance performance on standardized tests. While prior studies draw similar conclusions (e.g. Breinholt and Jæger 2020; Dumais 2006; for a review see Jæger 2022), we would like to note that standardized tests are not necessarily culturally “blind,” and may, just like teachers, inadvertently reward interactional culture. Students who have been socialized more with the language or question‐style employed in standardized tests may perform better in these tests, even if they are academically not more competent.

Our findings provide little support for the cultural reproduction hypothesis. We only found small SES differences in the interactional cultural capital indicators. Accordingly, these differences could not explain SES disparities in performance. Instead, findings suggest that certain forms of interactional cultural capital relate to educational performance *alongside* family background. Moreover, students from advantaged SES backgrounds did not seem to reap more educational benefits from their interactional cultural capital. Rather, in line with the cultural mobility hypothesis, students' educational knowledge was more positively related to (teacher‐assessed) performance among students whose parents did not hold a BA degree than among students whose parent(s) did. Possibly, this latter group does not need knowledge of the educational system to perform well, as their parents can navigate the educational system for them. Conversely, students without tertiary‐educated parents can rely less on their parents' knowledge in this respect, making their performance more dependent on their *own* knowledge. Particularly for this subgroup, interventions around key educational transitions, such as in‐school orientation or mentoring programs, may offer valuable opportunities to enhance their knowledge of possible educational pathways. Interestingly, the relationship between educational knowledge and school performance did not vary by household income. Compared to parental education, household income is arguably less reflexive of parents' educational knowledge.

This study also knows limitations. First, our findings may be confounded by unobserved student or parental traits. Some previous studies tried to overcome this by using sibling data and/or longitudinal information on cultural capital (e.g., Breinholt and Jæger 2020, Gaddis 2013; Jæger 2011; Jæger and Møllegaard 2017). In our data, cultural capital was assessed only once. However, even with longitudinal measures, it is unlikely that students' cultural capital would substantially change between closely spaced waves. Moreover, within‐family or within‐student estimates risk overcontrolling. Since such models only use variation in cultural capital *within* a family or even a student, they cannot capture the effects of cultural capital acquired during (early) family socialization. However, precisely this type of cultural capital, which is difficult to acquire or convey later in life, is argued to be most relevant in educational settings (Edgerton and Roberts 2014; Lareau and Weininger 2003).

Second, while we tried to incorporate different expressions of interactional cultural capital, not all potentially relevant ones were included in the study (e.g., accents, bodily manifestations). This limitation arises partly because some expressions are hard to capture quantitatively. Relatedly, future studies may want to use more elaborate measures to capture the expressions studied here (e.g., more items/dimensions on educational knowledge), and could benefit from relying on dyadic data that does not only include the students' perspective but also teachers' interpretation of cultural capital signals. Such an approach would align better with the presumed interactional nature of this type of cultural capital.

Related to the difficulties of measuring cultural capital is its ongoing conceptual ambiguity. Scholars vary in their cultural capital definition and also in how they relate it to other important concepts, such as habitus. Some scholars describe embodied cultural capital and habitus as “moments of the same thing” (Edgerton and Roberts 2014, 207, 212): in certain fields, interactional dispositions can have value and act as a resource. However, other scholars make a slightly sharper distinction between the two, describing cultural capital as high‐status cultural *signals* (i.e., external), and habitus as *internalized* class‐based dispositions and perceptions (Dumais 2006; Bodovski 2014). Our conceptualization aligned more with the former: we considered students' educational knowledge and sense of ease in school as aspect of cultural capital, assuming these dispositions hold value in the school context (Edgerton and Roberts 2014, 207). However, we are aware that others may classify them as habitus‐related aspects, reflecting students' internalized perceptions of the educational context.

Overall, our study shows that specific manifestations of children's *own* interactional capital positively relate to educational outcomes, above and beyond associations with parental education and students' prior performance. We hope this initial effort to quantify various forms of interactional cultural capital, and their relation with academic performance, paves the way for future (quantitative) studies to expand conceptualizations of cultural capital in educational settings. To better understand how cultural capital is acquired and converted into educational benefits, a broader scope—beyond elite cultural consumption—is warranted.

## Supporting information

Supporting Information S1

## Data Availability

The data that support the findings of this study are available on request from the corresponding author. The data are not publicly available due to privacy or ethical restrictions.

## Endnotes

^1^
This describes the procedure at the time of data collection. From 2023–2024 onwards, upward adjustments occur in all cases, except when a school makes an explicit motivated objection.

^2^
To compare scores across different tests, we calculate standardized percentile scores (i.e., standardization of the percentage of all students with the same score or lower on the same test). In the full population, the standard‐normal scores for all five tests follow a normal distribution (mean 0, standard deviation 1).

^3^
Children with a migration background from Europe (excl. Turkey), the United States, Oceania, Indonesia, or Japan are considered to be more similar to children without a migration background than to children with a migration background from other countries (Vermeij, Van Duijn, and Baerveldt 2009).

^4^
SUR estimates replicate those from multivariate models when the same predictors are used for both outcomes. We use SURs, as our control variables differ across the two equations (i.e., we control for the type of test for the test‐based assessment).

^5^
For the full sample, we also estimate a model including all three sets. Conclusions remain the same (Supporting Information S1: Table A3, model 8).

^6^
We also estimated multi‐level multivariate models accounting for the nesting of students in schools, using Baldwin et al. (2014)'s approach in Stata. These models are computationally demanding, and do not all converge when accounting for students' nesting in classes. As conclusions remain similar (Supporting Information S1: Figures A1‐ A2), we focus on SURs.
