# Supplementary material for: Students' Interactional Cultural Capital and Academic Performance in Test‐ and Teacher‐Based Assessments
Source: Br J Sociol. 2025 Mar 7;76(3):622–34. doi: 10.1111/1468-4446.13199 (PMC12163557; doi:10.1111/1468-4446.13199)
Supplement: Supplementary file 1 — Supporting Information S1 [file BJOS-76-622-s001.docx]

# **Appendix**

## **Tables**

Table A1: Sample descriptives

|  | Full sample:  *M* (*SD*) or % | Subsample:  *M* (*SD*) or % | *p*-value sig. test  of difference |
| --- | --- | --- | --- |
| Teacher-based assessment (std) | 0.00 (1.00) | -0.05 (0.99) | .27 |
| Test-based assessment (std) | 0.00 (1.00) | -0.08 (1.00) | .07 |
| Test type |  |  | <.01 |
| CET | 44.8% | 50.0% |  |
| IEP | 28.0% | 16.0% |  |
| Route8 | 18.8% | 20.7% |  |
| DIA | 5.1% | 2.5% |  |
| AMN | 3.2% | 10.8% |  |
| Gender (girl = 1) | 51.4% | 53.3% | .41 |
| Migration background (yes = 1) | 9.8% | 10.5% | .66 |
| Age | 11.74 (0.43) | 11.72 (0.40) | .29 |
| Parental education (BA degree = 1) | 53.3% | 55.8% | .28 |
| Difference home-school | 1.62 (0.95) | 1.60 (1.00) | .61 |
| Educational knowledge | 1.54 (1.46) | 1.52 (1.41) | .77 |
| Assertive help | 2.32 (0.96) | 2.44 (0.98) | < .01 |
| Test help | 2.65 (1.05) | 2.65 (1.05) | .90 |
| Affirmative help | 2.76 (0.99) | 2.69 (0.96) | .11 |
| Public help | 2.43 (1.11) | 2.37 (1.11) | .22 |
| *N* | 1,248 | 362 |  |

*Note*: The statistical significance of differences between the sample and the subsample is assessed with a t-test (for continuous variables), z-test (for binary variables) or Chi-Squared test (for categorical variables with over two categories).

**Table A2: Correlations for study variables**

|  | 1 | 2 | 3 | 4 | 5 | 6 | 7 | 9 | 9 | 10 | 11 |
| --- | --- | --- | --- | --- | --- | --- | --- | --- | --- | --- | --- |
| 1. Teacher-based assessment (std) |  |  |  |  |  |  |  |  |  |  |  |
| 2. Test-based assessment (std) | 0.81^***^ |  |  |  |  |  |  |  |  |  |  |
| 3. Gender | -0.06^*^ | -0.03 |  |  |  |  |  |  |  |  |  |
| 4. Migration background | -0.01 | 0.00 | 0.05 |  |  |  |  |  |  |  |  |
| 5. Age | -0.32^***^ | -0.26^***^ | -0.06^*^ | 0.09^**^ |  |  |  |  |  |  |  |
| 6. Parental education | 0.38^***^ | 0.32^***^ | -0.04 | -0.10^***^ | -0.18^***^ |  |  |  |  |  |  |
| 7. Diff. home-school | -0.15^***^ | -0.17^***^ | -0.13^***^ | 0.06^*^ | 0.06^*^ | -0.09^***^ |  |  |  |  |  |
| 8. Edu. knowledge | 0.23^***^ | 0.21^***^ | -0.05 | 0.07^*^ | -0.10^***^ | 0.11^***^ | -0.06^*^ |  |  |  |  |
| 9. Assertive help | 0.00 | 0.00 | -0.05 | -0.03 | -0.04 | -0.06^*^ | -0.01 | 0.03 |  |  |  |
| 10. Test help | 0.07^*^ | 0.08^**^ | 0.10^***^ | -0.03 | -0.02 | -0.03 | -0.08^**^ | -0.03 | 0.21^***^ |  |  |
| 11. Affirmative help | 0.10^***^ | 0.10^***^ | 0.04 | 0.01 | 0.00 | -0.01 | -0.11^***^ | 0.05 | 0.26^***^ | 0.32^***^ |  |
| 12. Public help | -0.05 | -0.10^***^ | 0.01 | -0.01 | 0.06^*^ | -0.06^*^ | -0.04 | 0.03 | 0.23^***^ | 0.25^***^ | 0.19^***^ |

Note: *N* = 1,248. ^*^*p* < .05, ^**^*p* < .01, ^***^*p* < .001.

**Table A3: SUR estimates of teacher- and test-based assessment of student performance, full sample**

|  | (1) | (2) | (3) | (4) | (5) | (6) | (7) | (8) |
| --- | --- | --- | --- | --- | --- | --- | --- | --- |
| **DV: Test-based assessment** |  |  |  |  |  |  |  |  |
| Parent with BA degree | 0.570*** | 0.545*** | 0.537*** | 0.563*** | 0.544*** | 0.534*** | 0.561*** | 0.509 *** |
|  | (0.062) | (0.061) | (0.064) | (0.059) | (0.061) | (0.064) | (0.059) | (0.059) |
| Migration background | 0.166 | 0.191* | 0.119 | 0.163 | 0.184* | 0.101 | 0.154 | 0.141 |
|  | (0.094) | (0.091) | (0.092) | (0.091) | (0.092) | (0.093) | (0.092) | (0.086) |
| Girl | -0.074 | -0.110* | -0.058 | -0.095 | -0.110* | -0.067 | -0.095 | -0.111 * |
|  | (0.053) | (0.053) | (0.054) | (0.053) | (0.054) | (0.054) | (0.053) | (0.053) |
| Age (std.) | -0.216*** | -0.212*** | -0.202*** | -0.209*** | -0.212*** | -0.200*** | -0.212*** | -0.191 *** |
|  | (0.026) | (0.026) | (0.026) | (0.025) | (0.026) | (0.026) | (0.025) | (0.025) |
| Test type (ref. = CITO) |  |  |  |  |  |  |  |  |
| IEP | -0.019 | -0.018 | -0.021 | -0.019 | -0.015 | -0.021 | -0.019 | -0.020 |
|  | (0.094) | (0.093) | (0.095) | (0.094) | (0.093) | (0.095) | (0.095) | (0.093) |
| Route 8 | -0.125 | -0.125 | -0.124 | -0.134 | -0.125 | -0.124 | -0.131 | -0.135 |
|  | (0.082) | (0.081) | (0.081) | (0.081) | (0.081) | (0.081) | (0.081) | (0.080) |
| DIA | 0.092 | 0.087 | 0.090 | 0.083 | 0.078 | 0.095 | 0.086 | 0.076 |
|  | (0.093) | (0.096) | (0.093) | (0.093) | (0.095) | (0.093) | (0.092) | (0.095) |
| AMN | 0.194** | 0.185** | 0.192** | 0.202*** | 0.190** | 0.190** | 0.204*** | 0.193 *** |
|  | (0.059) | (0.059) | (0.061) | (0.056) | (0.058) | (0.064) | (0.057) | (0.058) |
| Dif. home-school (std.) |  | -0.137*** |  |  | -0.181*** |  |  | -0.120 *** |
|  |  | (0.027) |  |  | (0.038) |  |  | (0.028) |
| Parent BA degree*Dif. home-sch |  |  |  |  | 0.085 |  |  |  |
|  |  |  |  |  | (0.049) |  |  |  |
| Edu knowledge (std.) |  |  | 0.157*** |  |  | 0.225*** |  | 0.154 *** |
|  |  |  | (0.028) |  |  | (0.037) |  | (0.028) |
| Parent BA degree*Edu knowl. |  |  |  |  |  | -0.120* |  |  |
|  |  |  |  |  |  | (0.057) |  |  |
| Assertive help (std.) |  |  |  | -0.014 |  |  | -0.057 | -0.014 |
|  |  |  |  | (0.026) |  |  | (0.040) | (0.026) |
| Parent BA degree*Assert. help |  |  |  |  |  |  | 0.078 |  |
|  |  |  |  |  |  |  | (0.060) |  |
| Test help (std.) |  |  |  | 0.079* |  |  | 0.064 | 0.085 * |
|  |  |  |  | (0.034) |  |  | (0.060) | (0.034) |
| Parent BA degree*Test. help |  |  |  |  |  |  | 0.025 |  |
|  |  |  |  |  |  |  | (0.073) |  |
| Affirmative help (std.) |  |  |  | 0.102*** |  |  | 0.147** | 0.081 ** |
|  |  |  |  | (0.028) |  |  | (0.048) | (0.027) |
| Parent BA degree*Affirm. help |  |  |  |  |  |  | -0.080 |  |
|  |  |  |  |  |  |  | (0.064) |  |
| Public help (std.) |  |  |  | -0.107*** |  |  | -0.099** | -0.117 *** |
|  |  |  |  | (0.030) |  |  | (0.037) | (0.029) |
| Parent BA degree*Public help |  |  |  |  |  |  | -0.013 |  |
|  |  |  |  |  |  |  | (0.053) |  |
| **DV: Teacher-based assessment** |  |  |  |  |  |  |  |  |
| Parent with BA degree | 0.657*** | 0.636*** | 0.623*** | 0.655*** | 0.635*** | 0.617*** | 0.655*** |  |
|  | (0.061) | (0.060) | (0.060) | (0.058) | (0.060) | (0.059) | (0.059) |  |
| Migration background | 0.170 | 0.192* | 0.121 | 0.170 | 0.189* | 0.091 | 0.163 |  |
|  | (0.099) | (0.095) | (0.097) | (0.097) | (0.095) | (0.098) | (0.099) |  |
| Girl | -0.130* | -0.161** | -0.113* | -0.149** | -0.161** | -0.129* | -0.148* |  |
|  | (0.057) | (0.056) | (0.058) | (0.057) | (0.057) | (0.057) | (0.058) |  |
| Age (std.) | -0.271*** | -0.268*** | -0.256*** | -0.268*** | -0.267*** | -0.253*** | -0.269*** |  |
|  | (0.026) | (0.026) | (0.025) | (0.026) | (0.026) | (0.025) | (0.026) |  |
| Dif. home-school (std.) |  | -0.118*** |  |  | -0.137*** |  |  |  |
|  |  | (0.027) |  |  | (0.039) |  |  |  |
| Parent BA degree*Dif. home-sch |  |  |  |  | 0.036 |  |  |  |
|  |  |  |  |  | (0.048) |  |  |  |
| Edu knowledge (std.) |  |  | 0.165*** |  |  | 0.280*** |  |  |
|  |  |  | (0.026) |  |  | (0.040) |  |  |
| Parent BA degree*Edu knowl. |  |  |  |  |  | -0.204*** |  |  |
|  |  |  |  |  |  | (0.054) |  |  |
| Assertive help (std.) |  |  |  | -0.018 |  |  | -0.030 |  |
|  |  |  |  | (0.027) |  |  | (0.042) |  |
| Parent BA degree*Assert. help |  |  |  |  |  |  | 0.021 |  |
|  |  |  |  |  |  |  | (0.054) |  |
| Test help (std.) |  |  |  | 0.062* |  |  | 0.078 |  |
|  |  |  |  | (0.031) |  |  | (0.056) |  |
| Parent BA degree*Test. help |  |  |  |  |  |  | -0.031 |  |
|  |  |  |  |  |  |  | (0.063) |  |
| Affirmative help (std.) |  |  |  | 0.094*** |  |  | 0.126* |  |
|  |  |  |  | (0.028) |  |  | (0.051) |  |
| Parent BA degree*Affirm. help |  |  |  |  |  |  | -0.062 |  |
|  |  |  |  |  |  |  | (0.063) |  |
| Public help (std.) |  |  |  | -0.040 |  |  | -0.045 |  |
|  |  |  |  | (0.028) |  |  | (0.038) |  |
| Parent BA degree*Public help |  |  |  |  |  |  | 0.011 |  |
|  |  |  |  |  |  |  | (0.048) |  |
| N | 1248 | 1248 | 1248 | 1248 | 1248 | 1248 | 1248 |  |

*** p < 0.001; ** p < 0.01; * p < 0.05.

**Table A4: SUR estimates of teacher- and test-based assessment of student performance, subsample**

|  | | (1) | | (2) | | (3) | | (4) | (5) | (6) | (7) |
| --- | --- | --- | --- | --- | --- | --- | --- | --- | --- | --- | --- |
| **DV: Test-based assessment** | |  | |  | |  | |  |  |  |  |
| Parent with BA degree | | 0.514*** | | 0.476*** | | 0.480*** | | 0.540*** | 0.475*** | 0.481*** | 0.502*** |
|  | | (0.110) | | (0.102) | | (0.112) | | (0.106) | (0.103) | (0.112) | (0.107) |
| Migration background | | -0.047 | | -0.017 | | -0.090 | | -0.043 | -0.025 | -0.093 | -0.055 |
|  | | (0.209) | | (0.194) | | (0.200) | | (0.210) | (0.195) | (0.202) | (0.208) |
| Girl | | -0.006 | | -0.059 | | 0.009 | | -0.034 | -0.056 | 0.009 | -0.027 |
|  | | (0.108) | | (0.108) | | (0.105) | | (0.114) | (0.108) | (0.105) | (0.114) |
| Age (std.) | | -0.192*** | | -0.200*** | | -0.172*** | | -0.196*** | -0.199*** | -0.172*** | -0.199*** |
|  | | (0.044) | | (0.046) | | (0.045) | | (0.044) | (0.046) | (0.045) | (0.046) |
| Test type (ref. = CITO) | |  | |  | |  | |  |  |  |  |
| IEP | | 0.015 | | 0.016 | | 0.018 | | 0.020 | 0.018 | 0.012 | 0.026 |
|  | | (0.124) | | (0.123) | | (0.121) | | (0.119) | (0.124) | (0.121) | (0.117) |
| Route 8 | | 0.007 | | 0.010 | | 0.007 | | 0.016 | 0.012 | 0.009 | 0.034 |
|  | | (0.144) | | (0.144) | | (0.143) | | (0.149) | (0.143) | (0.145) | (0.144) |
| DIA | | 0.347* | | 0.335* | | 0.326 | | 0.310* | 0.328 | 0.348* | 0.287 |
|  | | (0.153) | | (0.167) | | (0.182) | | (0.151) | (0.170) | (0.157) | (0.159) |
| AMN | | 0.297** | | 0.294** | | 0.290* | | 0.302** | 0.296** | 0.284* | 0.312** |
|  | | (0.107) | | (0.108) | | (0.114) | | (0.102) | (0.108) | (0.120) | (0.103) |
| Dif. home-school (std.) | |  | | -0.153** | |  | |  | -0.185* |  |  |
|  | |  | | (0.052) | |  | |  | (0.074) |  |  |
| Parent BA degree*Dif. home-sch | |  | |  | |  | |  | 0.055 |  |  |
|  | |  | |  | |  | |  | (0.090) |  |  |
| Edu knowledge (std.) | |  | |  | | 0.174** | |  |  | 0.175* |  |
|  | |  | |  | | (0.055) | |  |  | (0.076) |  |
| Parent BA degree*Edu knowl. | |  | |  | |  | |  |  | -0.002 |  |
|  | |  | |  | |  | |  |  | (0.118) |  |
| Assertive help (std.) | |  | |  | |  | | 0.064 |  |  | -0.025 |
|  | |  | |  | |  | | (0.045) |  |  | (0.068) |
| Parent BA degree*Assert. help | |  | |  | |  | |  |  |  | 0.163 |
|  | |  | |  | |  | |  |  |  | (0.112) |
| Test help (std.) | |  | |  | |  | | 0.114* |  |  | 0.090 |
|  | |  | |  | |  | | (0.055) |  |  | (0.080) |
| Parent BA degree*Test. help | |  | |  | |  | |  |  |  | 0.038 |
|  | |  | |  | |  | | 0.092 |  |  | (0.123) |
| Affirmative help (std.) | |  | |  | |  | | (0.057) |  |  | 0.178* |
|  | |  | |  | |  | |  |  |  | (0.079) |
| Parent BA degree*Affirm. help | |  | |  | |  | |  |  |  | -0.165 |
|  | |  | |  | |  | | -0.033 |  |  | (0.101) |
| Public help (std.) | |  | |  | |  | | (0.047) |  |  | -0.004 |
|  | |  | |  | |  | |  |  |  | (0.063) |
|  | |  | |  | |  | |  |  |  |  |
| Parent BA degree*Public help | |  | |  | |  | |  |  |  | -0.062 |
|  | |  | |  | |  | |  |  |  | (0.102) |
| **DV: Teacher-based assessment** | |  | |  | |  | |  |  |  |  |
| Parent with BA degree | | 0.536*** | | 0.495*** | | 0.506*** | | 0.562*** | 0.495*** | 0.501*** | 0.542*** |
|  | | (0.100) | | (0.092) | | (0.097) | | (0.096) | (0.092) | (0.099) | (0.094) |
| Migration background | | -0.025 | | 0.005 | | -0.064 | | -0.027 | -0.001 | -0.074 | -0.024 |
|  | | (0.209) | | (0.185) | | (0.206) | | (0.212) | (0.183) | (0.210) | (0.217) |
| Girl | | -0.111 | | -0.169 | | -0.099 | | -0.135 | -0.167 | -0.112 | -0.134 |
|  | | (0.115) | | (0.116) | | (0.111) | | (0.118) | (0.116) | (0.110) | (0.119) |
| Age (std.) | | -0.216*** | | -0.225*** | | -0.199*** | | -0.223*** | -0.224*** | -0.196*** | -0.224*** |
|  | | (0.046) | | (0.050) | | (0.048) | | (0.047) | (0.050) | (0.047) | (0.049) |
| Dif. home-school (std.) | |  | | -0.164** | |  | |  | -0.187 |  |  |
|  | |  | | (0.051) | |  | |  | (0.096) |  |  |
| Parent BA degree*Dif. home-sch | |  | |  | |  | |  | 0.039 |  |  |
|  | |  | |  | |  | |  | (0.123) |  |  |
| Edu knowledge (std.) | |  | |  | | 0.149* | |  |  | 0.233* |  |
|  | |  | |  | | (0.060) | |  |  | (0.090) |  |
| Parent BA degree*Edu knowl. | |  | |  | |  | |  |  | -0.157 |  |
|  | |  | |  | |  | |  |  | (0.122) |  |
| Assertive help (std.) | |  | |  | |  | | 0.028 |  |  | -0.033 |
|  | |  | |  | |  | | (0.052) |  |  | (0.091) |
| Parent BA degree*Assert. help | |  | |  | |  | |  |  |  | 0.111 |
|  | |  | |  | |  | |  |  |  | (0.120) |
| Test help (std.) | |  | |  | |  | | 0.063 |  |  | 0.070 |
|  | |  | |  | |  | | (0.058) |  |  | (0.094) |
| Parent BA degree*Test. help | |  | |  | |  | |  |  |  | -0.015 |
|  | |  | |  | |  | |  |  |  | (0.130) |
| Affirmative help (std.) | |  | |  | |  | | 0.100 |  |  | 0.139 |
|  | |  | |  | |  | | (0.062) |  |  | (0.117) |
| Parent BA degree*Affirm. help | |  | |  | |  | |  |  |  | -0.076 |
|  | |  | |  | |  | |  |  |  | (0.123) |
| Public help (std.) | |  | |  | |  | | 0.055 |  |  | 0.056 |
|  | |  | |  | |  | | (0.051) |  |  | (0.089) |
| Parent BA degree*Public help | |  | |  | |  | |  |  |  | -0.005 |
|  | |  | |  | |  | |  |  |  | (0.120) |
| N | | 362 | | 362 | | 362 | | 362 | 362 | 362 | 362 |
|  |  | |  | |  | |  |  |  |  |  |

*** p < 0.001; ** p < 0.01; * p < 0.05.

**Table A5: SUR estimates of teacher- and test-based assessment of student performance, accounting for student performance in grade 5 (t-1)**

|  | (1) | | (2) | | (3) | | (4) | | (5) | | (6) | | (7) | |  |
| --- | --- | --- | --- | --- | --- | --- | --- | --- | --- | --- | --- | --- | --- | --- | --- |
| **DV: Test-based assessment** |  | |  | |  | |  |  |  |  |  |  |  |  |  |
| Parent with BA degree | 0.134* | | 0.117 | | 0.111 | | 0.154* | | 0.117 | | 0.113 | | 0.138* | |  |
|  | (0.068) | | (0.063) | | (0.068) | | (0.066) | | (0.064) | | (0.067) | | (0.068) | |  |
| Migration background | 0.097 | | 0.113 | | 0.063 | | 0.096 | | 0.106 | | 0.065 | | 0.077 | |  |
|  | (0.125) | | (0.120) | | (0.118) | | (0.122) | | (0.122) | | (0.117) | | (0.124) | |  |
| Girl | 0.186** | | 0.154* | | 0.198** | | 0.173* | | 0.157** | | 0.205** | | 0.180* | |  |
|  | (0.067) | | (0.060) | | (0.064) | | (0.072) | | (0.058) | | (0.064) | | (0.072) | |  |
| Age (std.) | -0.034 | | -0.041 | | -0.020 | | -0.037 | | -0.040 | | -0.021 | | -0.040 | |  |
|  | (0.026) | | (0.027) | | (0.027) | | (0.026) | | (0.026) | | (0.027) | | (0.027) | |  |
| Test type (ref. = CITO) |  | |  | |  | |  | |  | |  | |  | |  |
| IEP | 0.005 | | 0.006 | | 0.008 | | 0.009 | | 0.008 | | 0.003 | | 0.013 | |  |
|  | (0.106) | | (0.104) | | (0.101) | | (0.104) | | (0.105) | | (0.102) | | (0.101) | |  |
| Route 8 | 0.028 | | 0.033 | | 0.029 | | 0.034 | | 0.036 | | 0.030 | | 0.047 | |  |
|  | (0.130) | | (0.129) | | (0.125) | | (0.134) | | (0.129) | | (0.126) | | (0.129) | |  |
| DIA | 0.394** | | 0.376** | | 0.364* | | 0.348** | | 0.365* | | 0.383** | | 0.325* | |  |
|  | (0.127) | | (0.145) | | (0.159) | | (0.131) | | (0.147) | | (0.138) | | (0.139) | |  |
| AMN | 0.313** | | 0.309** | | 0.303** | | 0.310*** | | 0.311** | | 0.297** | | 0.314*** | |  |
|  | (0.095) | | (0.095) | | (0.103) | | (0.093) | | (0.096) | | (0.109) | | (0.093) | |  |
| Read score gr. 5 | 0.323*** | | 0.318*** | | 0.314*** | | 0.309*** | | 0.316*** | | 0.315*** | | 0.306*** | |  |
|  | (0.048) | | (0.049) | | (0.044) | | (0.049) | | (0.048) | | (0.044) | | (0.050) | |  |
| Math score gr. 5 | 0.530*** | | 0.525*** | | 0.530*** | | 0.530*** | | 0.526*** | | 0.531*** | | 0.531*** | |  |
|  | (0.047) | | (0.046) | | (0.044) | | (0.048) | | (0.045) | | (0.044) | | (0.050) | |  |
| Dif. home-school (std.) |  | | -0.086** | |  | |  | | -0.115** | |  | |  | |  |
|  |  | | (0.029) | |  | |  | | (0.042) | |  | |  | |  |
| Parent BA degree*Dif. home-sch |  | |  | |  | |  | | 0.050 | |  | |  | |  |
|  |  | |  | |  | |  | | (0.064) | |  | |  | |  |
| Edu knowledge (std.) |  | |  | | 0.135*** | |  | |  | | 0.099* | |  | |  |
|  |  | |  | | (0.039) | |  | |  | | (0.048) | |  | |  |
| Parent BA degree*Edu knowl. |  | |  | |  | |  | |  | | 0.068 | |  | |  |
|  |  | |  | |  | |  | |  | | (0.074) | |  | |  |
| Assertive help (std.) |  | |  | |  | | 0.057* | |  | |  | | 0.038 | |  |
|  |  | |  | |  | | (0.025) | |  | |  | | (0.040) | |  |
| Parent BA degree*Assert. help |  | |  | |  | |  | |  | |  | | 0.037 | |  |
|  |  | |  | |  | |  | |  | |  | | (0.075) | |  |
| Test help (std.) |  | |  | |  | | 0.058 | |  | |  | | 0.027 | |  |
|  |  | |  | |  | | (0.043) | |  | |  | | (0.048) | |  |
| Parent BA degree*Test. help |  | |  | |  | |  | |  | |  | | 0.052 | |  |
|  |  | |  | |  | |  | |  | |  | | (0.070) | |  |
| Affirmative help (std.) |  | |  | |  | | 0.064 | |  | |  | | 0.112* | |  |
|  |  | |  | |  | | (0.039) | |  | |  | | (0.044) | |  |
| Parent BA degree*Affirm. help |  | |  | |  | |  | |  | |  | | -0.092 | |  |
|  | |  | |  | |  | |  | |  | |  | | (0.066) | |

| Public help (std.) | |  | |  | |  | | -0.040 |  |  | -0.004 |  |
| --- | --- | --- | --- | --- | --- | --- | --- | --- | --- | --- | --- | --- |
|  | |  | |  | |  | | (0.029) |  |  | (0.043) |  |
| Parent BA degree*Public help | |  | |  | |  | |  |  |  | -0.071 |  |
|  | |  | |  | |  | |  |  |  | (0.061) |  |
| **DV: Teacher-based assessment** | |  | |  | |  | | | | | | |
| Parent with BA degree | | 0.145* | | 0.127* | | 0.127* | | 0.162** | 0.127* | 0.125* | 0.163** |  |
|  | | (0.061) | | (0.060) | | (0.056) | | (0.055) | (0.060) | (0.057) | (0.052) |  |
| Migration background | | 0.135 | | 0.150 | | 0.105 | | 0.127 | 0.145 | 0.099 | 0.125 |  |
|  | | (0.109) | | (0.094) | | (0.107) | | (0.107) | (0.094) | (0.109) | (0.112) |  |
| Girl | | 0.050 | | 0.015 | | 0.060 | | 0.042 | 0.017 | 0.052 | 0.043 |  |
|  | | (0.079) | | (0.077) | | (0.075) | | (0.084) | (0.078) | (0.075) | (0.085) |  |
| Age (std.) | | -0.055 | | -0.063 | | -0.044 | | -0.061 | -0.062 | -0.042 | -0.061 |  |
|  | | (0.033) | | (0.035) | | (0.033) | | (0.034) | (0.035) | (0.033) | (0.035) |  |
| Read score gr. 5 | | 0.392*** | | 0.387*** | | 0.386*** | | 0.383*** | 0.386*** | 0.385*** | 0.383*** |  |
|  | | (0.045) | | (0.045) | | (0.041) | | (0.045) | (0.045) | (0.040) | (0.045) |  |
| Math score gr. 5 | | 0.481*** | | 0.475*** | | 0.481*** | | 0.481*** | 0.475*** | 0.479*** | 0.481*** |  |
|  | | (0.043) | | (0.043) | | (0.042) | | (0.045) | (0.042) | (0.042) | (0.047) |  |
| Dif. home-school (std.) | |  | | -0.096** | |  | |  | -0.112** |  |  |  |
|  | |  | | (0.034) | |  | |  | (0.035) |  |  |  |
| Parent BA degree*Dif. home-sch | |  | |  | |  | |  | 0.028 |  |  |  |
|  | |  | |  | |  | |  | (0.060) |  |  |  |
| Edu knowledge (std.) | |  | |  | | 0.109** | |  |  | 0.156** |  |  |
|  | |  | |  | | (0.036) | |  |  | (0.048) |  |  |
| Parent BA degree*Edu knowl. | |  | |  | |  | |  |  | -0.087 |  |  |
|  | |  | |  | |  | |  |  | (0.073) |  |  |
| Assertive help (std.) | |  | |  | |  | | 0.019 |  |  | 0.026 |  |
|  | |  | |  | |  | | (0.032) |  |  | (0.049) |  |
| Parent BA degree*Assert. help | |  | |  | |  | |  |  |  | -0.012 |  |
|  | |  | |  | |  | |  |  |  | (0.068) |  |
| Test help (std.) | |  | |  | |  | | 0.002 |  |  | 0.003 |  |
|  | |  | |  | |  | | (0.044) |  |  | (0.052) |  |
| Parent BA degree*Test. help | |  | |  | |  | |  |  |  | -0.000 |  |
|  | |  | |  | |  | |  |  |  | (0.070) |  |
| Affirmative help (std.) | |  | |  | |  | | 0.072 |  |  | 0.065 |  |
|  | |  | |  | |  | | (0.040) |  |  | (0.059) |  |
| Parent BA degree*Affirm. help | |  | |  | |  | |  |  |  | 0.010 |  |
|  | |  | |  | |  | |  |  |  | (0.079) |  |
| Public help (std.) | |  | |  | |  | | 0.047 |  |  | 0.059 |  |
|  | |  | |  | |  | | (0.038) |  |  | (0.061) |  |
| Parent BA degree*Public help | |  | |  | |  | |  |  |  | -0.020 |  |
|  | |  | |  | |  | |  |  |  | (0.082) |  |
| N | | 362 | | 362 | | 362 | | 362 | 362 | 362 | 362 |  |
|  |  | |  | |  | |  |  |  |  |  |  |

*** p < 0.001; ** p < 0.01; * p < 0.05.

## **Description of supplementary analyses**

We conduct several robustness checks to corroborate our main findings.

First, we test for interactions between students’ perceived home-school differences on the one hand, and their educational knowledge or help-seeking behaviors on the other hand (see page 18). Harvey (2022) distinguishes between *what* culture is enacted and *how* this culture is enacted. While the ‘what’ refers to cognitive forms of culture, such as skills, practices, attitudes, preferences or knowing *what* is valued in the educational system; the ‘how’ refers to embodied cultural capital, such as mannerisms that allow students to successfully perform their cognitive culture. This cultural ‘how’ is often defined as a sense of ease in a particular context. Importantly, knowing *that* certain behaviors relate to educational benefits may not be sufficient to reap their full benefits. For example, children may know *that* they should ask for help, yet lack embodied culture that enables them to ask for help in a manner that leads to educational rewards. Accordingly, educational advantages related to knowing *what* it takes to be successful in school may be conditional on knowing *how* to interact and negotiate with educational gatekeepers. To test this, we interact students’ perceived home-school differences (i.e. as a measure of cultural *how)* with students’ educational knowledge in one model (table A6, model 1), and their help seeking behaviors in another model (table A6, model 2). We find no statistically significant interaction effects (joint test that the interaction effect(s) are zero for teacher- and test-based assessments of performance for educational knowledge: χ^2^(2)=1.52, *p*=0.47, and help-seeking behaviors χ^2^(8)=3.80, *p*=0.87).

Second, we estimate models on the full sample using different measures of student SES. First, we predict models in which we distinguish between three parental education categories (i.e., no BA degree, a BA degree, or an MA degree or higher) (see table A7). The main conclusions remain the same. Compared to students whose parents do not hold a BA degree, both students with (a) parent(s) with a BA and an MA degree show (1) higher performance in teacher- and test-based assessments; and (2) a weaker positive association between educational knowledge and (teacher-assessed) performance (model 4). The school performance of students whose parent(s) hold an MA degree is higher than that of students whose parent(s) hold a BA degree. However, the relation between educational knowledge and the performance outcomes does not significantly differ between these two groups (model 4).

In a second set of analyses, we use household income as a measure of student SES (see table A8). The findings are similar as the findings using parental education, except that we find no statistically significant interaction between household income and educational knowledge (model 4). Hence, the positive relation between educational knowledge and student performance does not vary by household income. Moreover, household income is equally positively related to performance in teacher- as well as test-based assessments of performance.

We estimate multilevel models – with students nested in classrooms –predicting teacher-assessed performance while accounting for test-assessed performance (table A9), and predicting test-assessed performance while accounting for teacher-assessed performance (table A10). In these models, we account for two school-level variables: the share of students with parent(s) with a BA-degree and the sixth grade students’ average score on the final test^[[1]](#footnote-1)^. See page 20.

Finally, as mentioned in footnote 4, we estimated multi-level multivariate models in Stata that account for the nesting of students in schools, using the approach discussed by Baldwin et al. (2014). Figure A1 and A2 display the main findings of these models.

**Table A6: SUR estimates of teacher and test-based assessments of student performance, including interactions for home-school difference with (1) eductional knowledge and (2) help-seeking.**

**Table A7: SUR estimates of teacher and test-based assessments of student performance using three parental education categories**

|  | | (1) | | (2) | | | (3) | | (4) | | (5) | |
| --- | --- | --- | --- | --- | --- | --- | --- | --- | --- | --- | --- | --- |
| **DV: Test-based assessment** | |  | | |  |  |  |  |  |  |  |  |
| ISCED 5-6 | 0.497*** | | 0.468*** | | | 0.467*** | | 0.495*** | | 0.463*** | |  |
|  | (0.065) | | (0.064) | | | (0.066) | | (0.064) | | (0.066) | |  |
| ISCED 7-8 | 0.709*** | | 0.691*** | | | 0.672*** | | 0.696*** | | 0.664*** | |  |
|  | (0.076) | | (0.075) | | | (0.077) | | (0.073) | | (0.078) | |  |
| Migration background | 0.152 | | 0.178 | | | 0.106 | | 0.152 | | 0.085 | |  |
|  | (0.096) | | (0.093) | | | (0.095) | | (0.094) | | (0.010) | |  |
| Girl | -0.071 | | -0.108* | | | -0.055 | | -0.094 | | -0.067 | |  |
|  | (0.051) | | (0.052) | | | (0.052) | | (0.051) | | (0.052) | |  |
| Age (std.) | -0.227*** | | -0.223*** | | | -0.212*** | | -0.220*** | | -0.211*** | |  |
|  | (0.026) | | (0.026) | | | (0.026) | | (0.025) | | (0.026) | |  |
| Test type (ref. = CITO) |  | |  | | |  | |  | |  | |  |
| IEP | -0.011 | | -0.010 | | | -0.012 | | -0.011 | | -0.013 | |  |
|  | (0.092) | | (0.091) | | | (0.093) | | (0.092) | | (0.093) | |  |
| Route 8 | -0.144 | | -0.144 | | | -0.143 | | -0.152 | | -0.144 | |  |
|  | (0.078) | | (0.078) | | | (0.078) | | (0.078) | | (0.078) | |  |
| DIA | 0.111 | | 0.106 | | | 0.109 | | 0.101 | | 0.109 | |  |
|  | (0.084) | | (0.085) | | | (0.085) | | (0.082) | | (0.086) | |  |
| AMN | 0.194*** | | 0.186** | | | 0.192** | | 0.202*** | | 0.194** | |  |
|  | (0.058) | | (0.058) | | | (0.060) | | (0.055) | | (0.061) | |  |
| Dif. home-school (std.) |  | | -0.138*** | | |  | |  | |  | |  |
|  |  | | (0.028) | | |  | |  | |  | |  |
| Edu knowledge (std.) |  | |  | | | 0.159*** | |  | | 0.241*** | |  |
|  |  | |  | | | (0.027) | |  | | (0.038) | |  |
| Edu knowledge (std.)*ISCED5-6 |  | |  | | |  | |  | | -0.177*** | |  |
|  |  | |  | | |  | |  | | (0.061) | |  |
| Edu knowledge (std.)*ISCED7-8 |  | |  | | |  | |  | | -0.103 | |  |
|  |  | |  | | |  | |  | | (0.069) | |  |
| Assertive help (std.) |  | |  | | |  | | -0.014 | |  | |  |
|  |  | |  | | |  | | (0.026) | |  | |  |
| Test help (std.) |  | |  | | |  | | 0.085** | |  | |  |
|  |  | |  | | |  | | (0.031) | |  | |  |
| Affirmative help (std.) |  | |  | | |  | | 0.095*** | |  | |  |
|  |  | |  | | |  | | (0.027) | |  | |  |
| Public help (std.) |  | |  | | |  | | -0.104*** | |  | |  |
|  |  | |  | | |  | | (0.030) | |  | |  |
| **DV: Teacher-based assessment** | | | | |  |  |  |  |  |  |  |  |
| ISCED 5-6 | 0.564*** | | 0.539*** | | | 0.533*** | | 0.567*** | | 0.527*** | |  |
|  | (0.067) | | (0.066) | | | (0.066) | | (0.066) | | (0.065) | |  |
| ISCED 7-8 | 0.801*** | | 0.785*** | | | 0.762*** | | 0.794*** | | 0.754*** | |  |
|  | (0.074) | | (0.073) | | | (0.076) | | (0.072) | | (0.075) | |  |
| Migration background | 0.166 | | 0.189* | | | 0.118 | | 0.167 | | 0.109 | |  |
|  | (0.098) | | (0.093) | | | (0.097) | | (0.096) | | (0.097) | |  |
| Girl | -0.127* | | -0.159** | | | -0.110 | | -0.146* | | -0.126 | |  |
|  | (0.056) | | (0.056) | | | (0.057) | | (0.057) | | (0.056) | |  |
| Age (std.) | -0.267*** | | -0.264*** | | | -0.252*** | | -0.265*** | | -0.250*** | |  |
|  | (0.026) | | (0.026) | | | (0.026) | | (0.026) | | (0.026) | |  |
| Dif. home-school (std.) |  | | -0.120*** | | |  | |  | |  | |  |
|  |  | | (0.028) | | |  | |  | |  | |  |
| Edu knowledge (std.) |  | |  | | | 0.164*** | |  | | 0.280*** | |  |
|  |  | |  | | | (0.026) | |  | | (0.040) | |  |
| Edu knowledge (std.)*ISCED5-6 |  | |  | | |  | |  | | -0.236** | |  |
|  |  | |  | | |  | |  | | (0.058) | |  |
| Edu knowledge (std.)*ISCED7-8 |  | |  | | |  | |  | | -0.168* | |  |
|  |  | |  | | |  | |  | | (0.069) | |  |
| Assertive help (std.) |  | |  | | |  | | -0.013 | |  | |  |
|  |  | |  | | |  | | (0.027) | |  | |  |
| Test help (std.) |  | |  | | |  | | 0.063* | |  | |  |
|  |  | |  | | |  | | (0.031) | |  | |  |
| Affirmative help (std.) |  | |  | | |  | | 0.090** | |  | |  |
|  |  | |  | | |  | | (0.028) | |  | |  |
| Public help (std.) |  | |  | | |  | | -0.040 | |  | |  |
|  |  | |  | | |  | | (0.028) | |  | |  |
| N | 1248 | | 1248 | | | 1248 | | 1248 | | 1248 | |  |

*Note:*** p < 0.001; ** p < 0.01; * p < 0.05.*

**Table A8: SUR estimates of teacher and test-based assessments of student performance using household income**

|  | (1) | (2) | | (3) | (4) | (5) |
| --- | --- | --- | --- | --- | --- | --- |
| **DV: Test-based assessment** | | |  |  |  |  |
| Household income (std.) | 0.130** | 0.120** | | 0.119** | 0.125** | 0.127** |
|  | (0.046) | (0.045) | | (0.042) | (0.045) | (0.064) |
| Migration bg | 0.190 | 0.216* | | 0.130 | 0.183 | 0.115 |
|  | (0.100) | (0.097) | | (0.097) | (0.098) | (0.098) |
| Girl | -0.096 | -0.137* | | -0.078 | -0.119* | -0.074 |
|  | (0.055) | (0.055) | | (0.055) | (0.055) | (0.055) |
| Age (std.) | -0.273*** | -0.267*** | | -0.254*** | -0.266*** | -0.251*** |
|  | (0.027) | (0.027) | | (0.026) | (0.027) | (0.026) |
| Test type (ref. = CITO) |  |  | |  |  |  |
| IEP | -0.013 | -0.011 | | -0.014 | -0.013 | -0.014 |
|  | (0.091) | (0.090) | | (0.091) | (0.091) | (0.091) |
| Route 8 | -0.142 | -0.142 | | -0.141 | -0.151 | -0.141 |
|  | (0.078) | (0.077) | | (0.077) | (0.078) | (0.077) |
| DIA | 0.105 | 0.100 | | 0.102 | 0.097 | 0.103 |
|  | (0.085) | (0.086) | | (0.086) | (0.082) | (0.087) |
| AMN | 0.191** | 0.182** | | 0.188** | 0.202*** | 0.188** |
|  | (0.063) | (0.062) | | (0.066) | (0.059) | (0.067) |
| Dif. home-school (std.) |  | -0.154*** | |  |  |  |
|  |  | (0.030) | |  |  |  |
| Edu knowledge (std.) |  |  | | 0.182*** |  | 0.182*** |
|  |  |  | | (0.029) |  | (0.029) |
| Edu knowledge * Hh income |  |  | |  |  | -0.044 |
|  |  |  | |  |  | (-0.029) |
| Assertive help (std.) |  |  | |  | -0.033 |  |
|  |  |  | |  | (0.029) |  |
| Test help (std.) |  |  | |  | 0.074* |  |
|  |  |  | |  | (0.032) |  |
| Affirmative help (std.) |  |  | |  | 0.103*** |  |
|  |  |  | |  | (0.029) |  |
| Public help (std.) |  |  | |  | -0.113*** |  |
|  |  |  | |  | (0.033) |  |
| **DV: Teacher-based assessment** | | |  |  |  |  |
| Household income (std.) | 0.133* | 0.124* | | 0.122* | 0.128* | 0.133* |
|  | (0.061) | (0.060) | | (0.056) | (0.061) | (0.061) |
| Migration background | 0.189 | 0.212* | | 0.125 | 0.181 | 0.104 |
|  | (0.108) | (0.104) | | (0.106) | (0.105) | (0.108) |
| Girl | -0.158** | -0.195** | | -0.139* | -0.178** | -0.133* |
|  | (0.060) | (0.059) | | (0.060) | (0.061) | (0.059) |
| Age (std.) | -0.318*** | -0.313*** | | -0.298*** | -0.316*** | -0.295*** |
|  | (0.030) | (0.030) | | (0.028) | (0.030) | (0.028) |
| Dif. home-school (std.) |  | -0.138*** | |  |  |  |
|  |  | (0.029) | |  |  |  |
| Edu knowledge (std.) |  |  | | 0.191*** |  | 0.191*** |
|  |  |  | | (0.029) |  | (0.028) |
|  |  |  | |  |  | -0.062 |
|  |  |  | |  |  | (0.044) |
| Assertive help (std.) |  |  | |  | -0.035 |  |
|  |  |  | |  | (0.030) |  |
| Test help (std.) |  |  | |  | 0.051 |  |
|  |  |  | |  | (0.033) |  |
| Affirmative help (std.) |  |  | |  | 0.099** |  |
|  |  |  | |  | (0.030) |  |
| Public help (std.) |  |  | |  | -0.049 |  |
|  |  |  | |  | (0.030) |  |
| N | 1240 | 1240 | | 1240 | 1240 | 1240 |
| *** p < 0.001; ** p < 0.01; * p < 0.05. | | |  |  |  |  |

**Table A9: Multi-level estimates of teacher-based assessment of student performance (track recommendations) while controlling for test-based assessment of student performance (final test score); students nested in classrooms**

|  | (1) | (2) | (3) | (4) | (5) | (6) | (7) |
| --- | --- | --- | --- | --- | --- | --- | --- |
|  | | | | | | | |
| Parent(s) with BA degree | 0.168^***^ | 0.165^***^ | 0.161^***^ | 0.169^***^ | 0.165^***^ | 0.160^***^ | 0.170^***^ |
|  | (0.033) | (0.034) | (0.033) | (0.033) | (0.034) | (0.033) | (0.034) |
|  |  |  |  |  |  |  |  |
| Final test score (std.) | 0.798^***^ | 0.794^***^ | 0.788^***^ | 0.798^***^ | 0.794^***^ | 0.785^***^ | 0.799^***^ |
|  | (0.018) | (0.018) | (0.018) | (0.018) | (0.018) | (0.018) | (0.018) |
|  |  |  |  |  |  |  |  |
| IEP | -0.070 | -0.071 | -0.072 | -0.068 | -0.072 | -0.071 | -0.067 |
|  | (0.066) | (0.068) | (0.068) | (0.068) | (0.068) | (0.067) | (0.068) |
|  |  |  |  |  |  |  |  |
| Route 8 | 0.051 | 0.050 | 0.051 | 0.065 | 0.050 | 0.050 | 0.063 |
|  | (0.074) | (0.076) | (0.076) | (0.076) | (0.077) | (0.076) | (0.076) |
|  |  |  |  |  |  |  |  |
| DIA | -0.099 | -0.104 | -0.104 | -0.103 | -0.101 | -0.123 | -0.110 |
|  | (0.106) | (0.109) | (0.109) | (0.109) | (0.109) | (0.108) | (0.109) |
|  |  |  |  |  |  |  |  |
| AMN | -0.071 | -0.076 | -0.078 | -0.084 | -0.079 | -0.067 | -0.083 |
|  | (0.161) | (0.167) | (0.166) | (0.167) | (0.168) | (0.166) | (0.167) |
|  |  |  |  |  |  |  |  |
| Girl | -0.069^*^ | -0.076^*^ | -0.064^*^ | -0.073^*^ | -0.076^*^ | -0.073^*^ | -0.072^*^ |
|  | (0.030) | (0.030) | (0.030) | (0.030) | (0.030) | (0.030) | (0.030) |
|  |  |  |  |  |  |  |  |
| Age (std.) | -0.110^***^ | -0.110^***^ | -0.107^***^ | -0.112^***^ | -0.110^***^ | -0.107^***^ | -0.110^***^ |
|  | (0.016) | (0.016) | (0.016) | (0.016) | (0.016) | (0.016) | (0.016) |
|  |  |  |  |  |  |  |  |
| Migration background | 0.011 | 0.016 | 0.003 | 0.010 | 0.017 | -0.014 | 0.011 |
|  | (0.052) | (0.052) | (0.052) | (0.052) | (0.052) | (0.052) | (0.052) |
|  |  |  |  |  |  |  |  |
| Difference home-school (std.) |  | -0.026 |  |  | -0.014 |  |  |
|  |  | (0.015) |  |  | (0.022) |  |  |
|  |  |  |  |  |  |  |  |
| Educational knowledge (std.) |  |  | 0.049^**^ |  |  | 0.112^***^ |  |
|  |  |  | (0.016) |  |  | (0.023) |  |
|  |  |  |  |  |  |  |  |
| Assertive help-seeking (std) |  |  |  | -0.008 |  |  | 0.010 |
|  |  |  |  | (0.016) |  |  | (0.024) |
|  |  |  |  |  |  |  |  |
| Test help-seeking (std) |  |  |  | 0.012 |  |  | 0.042 |
|  |  |  |  | (0.017) |  |  | (0.025) |
|  |  |  |  |  |  |  |  |
| Affirmative help-seeking (std) |  |  |  | 0.017 |  |  | 0.014 |
|  |  |  |  | (0.016) |  |  | (0.024) |
|  |  |  |  |  |  |  |  |
| Public help-seeking (std) |  |  |  | 0.037^*^ |  |  | 0.027 |
|  |  |  |  | (0.016) |  |  | (0.023) |
|  |  |  |  |  |  |  |  |
| Parent(s) BA degree*Dif. home-school (std.) |  |  |  |  | -0.023 |  |  |
|  |  |  |  |  | (0.030) |  |  |
|  |  |  |  |  |  |  |  |
| Parent(s) BA degree*Edu. know (std.) |  |  |  |  |  | -0.110^***^ |  |
|  |  |  |  |  |  | (0.030) |  |
|  |  |  |  |  |  |  |  |
| Parent(s) BA degree*Ass. help (std.) |  |  |  |  |  |  | -0.033 |
|  |  |  |  |  |  |  | (0.032) |
|  |  |  |  |  |  |  |  |
| Parent(s) BA degree*Test help (std.) |  |  |  |  |  |  | -0.054 |
|  |  |  |  |  |  |  | (0.033) |
|  |  |  |  |  |  |  |  |
| Parent(s) BA degree*Aff. help (std.) |  |  |  |  |  |  | 0.0002 |
|  |  |  |  |  |  |  | (0.033) |
|  |  |  |  |  |  |  |  |
| Parent(s) BA degree*Pub. help (std.) |  |  |  |  |  |  | 0.019 |
|  |  |  |  |  |  |  | (0.032) |
|  |  |  |  |  |  |  |  |
| **Level-2 variables** |  |  |  |  |  |  |  |
| Avg. test score school (std.) | -0.227^**^ | -0.229^**^ | -0.227^**^ | -0.227^**^ | -0.228^**^ | -0.236^**^ | -0.228^**^ |
|  | (0.079) | (0.082) | (0.081) | (0.082) | (0.082) | (0.081) | (0.081) |
| %Parent(s) with BA degree school | 0.292 | 0.294 | 0.306 | 0.314^*^ | 0.295 | 0.306 | 0.311^*^ |
|  | (0.152) | (0.158) | (0.157) | (0.158) | (0.158) | (0.156) | (0.158) |
|  | | | | | | | |
| Variance student-level | 0.047 | 0.053 | 0.052 | 0.052 | 0.053 | 0.051 | 0.052 |
| Variance class-level | 0.26 | 0.26 | 0.259 | 0.26 | 0.26 | 0.257 | 0.26 |
| N classes | 107 | 107 | 107 | 107 | 107 | 107 | 107 |
| N students | 1248 | 1248 | 1248 | 1248 | 1248 | 1248 | 1248 |
|  | | | | | | | |
| *Note:* | ^*^p<0.05; ^**^p<0.01; ^***^p<0.001 | | | | | | |

| **Table A10: Multi-level estimates of test-based assessment of student performance (final test score) while controlling for teacher-based assessment of student performance (track recommendation); students nested in classrooms** | | | | | | | |
| --- | --- | --- | --- | --- | --- | --- | --- |
|  | (1) | (2) | (3) | (4) | (5) | (6) | (7) |
|  | | | | | | | |
| Parent(s) with BA degree | 0.075^*^ | 0.072^*^ | 0.072^*^ | 0.075^*^ | 0.072^*^ | 0.071^*^ | 0.072^*^ |
|  | (0.034) | (0.034) | (0.034) | (0.034) | (0.034) | (0.034) | (0.034) |
|  |  |  |  |  |  |  |  |
| Teacher track recommendation (std.) | 0.771^***^ | 0.770^***^ | 0.769^***^ | 0.766^***^ | 0.769^***^ | 0.772^***^ | 0.766^***^ |
|  | (0.017) | (0.017) | (0.018) | (0.017) | (0.017) | (0.018) | (0.017) |
|  |  |  |  |  |  |  |  |
| IEP | 0.053 | 0.054 | 0.053 | 0.050 | 0.056 | 0.053 | 0.052 |
|  | (0.053) | (0.055) | (0.055) | (0.054) | (0.055) | (0.055) | (0.054) |
|  |  |  |  |  |  |  |  |
| Route 8 | -0.054 | -0.055 | -0.054 | -0.067 | -0.055 | -0.054 | -0.062 |
|  | (0.060) | (0.062) | (0.062) | (0.061) | (0.062) | (0.062) | (0.061) |
|  |  |  |  |  |  |  |  |
| DIA | 0.079 | 0.074 | 0.076 | 0.069 | 0.068 | 0.085 | 0.075 |
|  | (0.090) | (0.093) | (0.093) | (0.092) | (0.093) | (0.093) | (0.092) |
|  |  |  |  |  |  |  |  |
| AMN | 0.076 | 0.070 | 0.072 | 0.086 | 0.075 | 0.067 | 0.086 |
|  | (0.130) | (0.135) | (0.136) | (0.133) | (0.135) | (0.135) | (0.133) |
|  |  |  |  |  |  |  |  |
| Girl | 0.014 | 0.007 | 0.015 | 0.009 | 0.007 | 0.020 | 0.008 |
|  | (0.030) | (0.030) | (0.030) | (0.030) | (0.030) | (0.030) | (0.030) |
|  |  |  |  |  |  |  |  |
| Age (std.) | 0.009 | 0.008 | 0.010 | 0.011 | 0.009 | 0.010 | 0.009 |
|  | (0.016) | (0.016) | (0.016) | (0.016) | (0.016) | (0.016) | (0.016) |
|  |  |  |  |  |  |  |  |
| Migration background | 0.081 | 0.087 | 0.079 | 0.080 | 0.083 | 0.086 | 0.076 |
|  | (0.051) | (0.051) | (0.051) | (0.051) | (0.052) | (0.052) | (0.051) |
|  |  |  |  |  |  |  |  |
| Difference home-school (std.) |  | -0.023 |  |  | -0.044^*^ |  |  |
|  |  | (0.015) |  |  | (0.022) |  |  |
|  |  |  |  |  |  |  |  |
| Educational knowledge (std.) |  |  | 0.021 |  |  | -0.009 |  |
|  |  |  | (0.016) |  |  | (0.024) |  |
|  |  |  |  |  |  |  |  |
| Assertive help-seeking (std) |  |  |  | 0.003 |  |  | -0.030 |
|  |  |  |  | (0.016) |  |  | (0.023) |
|  |  |  |  |  |  |  |  |
| Test help-seeking (std) |  |  |  | 0.024 |  |  | -0.004 |
|  |  |  |  | (0.017) |  |  | (0.025) |
|  |  |  |  |  |  |  |  |
| Affirmative help-seeking (std) |  |  |  | 0.019 |  |  | 0.037 |
|  |  |  |  | (0.016) |  |  | (0.024) |
|  |  |  |  |  |  |  |  |
| Public help-seeking (std) |  |  |  | -0.068^***^ |  |  | -0.063^**^ |
|  |  |  |  | (0.016) |  |  | (0.023) |
|  |  |  |  |  |  |  |  |
| Avg. test score school (std.) | 0.550^***^ | 0.543^***^ | 0.546^***^ | 0.546^***^ | 0.542^***^ | 0.550^***^ | 0.547^***^ |
|  | (0.064) | (0.066) | (0.066) | (0.065) | (0.066) | (0.066) | (0.065) |
|  |  |  |  |  |  |  |  |
| %Parent(s) with BA degree school | -0.463^***^ | -0.451^***^ | -0.449^***^ | -0.473^***^ | -0.453^***^ | -0.449^***^ | -0.464^***^ |
|  | (0.126) | (0.131) | (0.131) | (0.129) | (0.131) | (0.131) | (0.129) |
|  |  |  |  |  |  |  |  |
| Parent(s) BA degree*Dif. home-school (std.) |  |  |  |  | 0.041 |  |  |
|  |  |  |  |  | (0.030) |  |  |
|  |  |  |  |  |  |  |  |
| Parent(s) BA degree*Edu. know (std.) |  |  |  |  |  | 0.053 |  |
|  |  |  |  |  |  | (0.031) |  |
|  |  |  |  |  |  |  |  |
| Parent(s) BA degree*Ass. help (std.) |  |  |  |  |  |  | 0.061 |
|  |  |  |  |  |  |  | (0.032) |
|  |  |  |  |  |  |  |  |
| Parent(s) BA degree*Test help (std.) |  |  |  |  |  |  | 0.047 |
|  |  |  |  |  |  |  | (0.032) |
|  |  |  |  |  |  |  |  |
| Parent(s) BA degree*Aff. help (std.) |  |  |  |  |  |  | -0.027 |
|  |  |  |  |  |  |  | (0.032) |
|  |  |  |  |  |  |  |  |
| Parent(s) BA degree*Pub. help (std.) |  |  |  |  |  |  | -0.009 |
|  |  |  |  |  |  |  | (0.031) |
|  |  |  |  |  |  |  |  |
| Constant | 0.230^**^ | 0.229^**^ | 0.224^**^ | 0.242^**^ | 0.232^**^ | 0.218^**^ | 0.240^**^ |
|  | (0.076) | (0.079) | (0.079) | (0.078) | (0.079) | (0.079) | (0.078) |
|  |  |  |  |  |  |  |  |
|  | | | | | | | |
| Variance student-level | 0.024 | 0.027 | 0.027 | 0.026 | 0.027 | 0.027 | 0.026 |
| Variance class-level | 0.261 | 0.262 | 0.262 | 0.26 | 0.262 | 0.262 | 0.259 |
| N classes | 107 | 107 | 107 | 107 | 107 | 107 | 107 |
| N students | 1248 | 1248 | 1248 | 1248 | 1248 | 1248 | 1248 |
|  | | | | | | | |
| *Note:* | ^*^p<0.05; ^**^p<0.01; ^***^p<0.001 | | | | | | |

**Figure A1: Multi-level multivariate estimates of teacher- and test-based assessment of student performance, students nested in schools**

**

*
Note: Estimates are based on models including all control variables. Top panel does not include controls for prior student performance (n=1248), bottom panel does include controls (n=362)*

**Figure A2: Interaction between educational knowledge and parental education, based on multi-level multivariate estimates of teacher- and test-based assessment of student performance**

*Note: Top-panel predictions are based on a model without controlling for prior student performance (n=1248), bottom-panel predictions are based on a of models accounting for prior student performance (n=362)*

1. The extent to which interactional forms of cultural capital are rewarded in school may dependent on a student’s specific teacher. Hence, using the same multi-level models, we also examined whether the effects of the cultural capital indicators varied across classrooms (i.e., tested for random slopes of the cultural capital variables). We do not find any statistically significant random slopes. [↑](#footnote-ref-1)
